# Rheological Optimization and Strength Development of Silica Fume-Modified All-Solid-Waste Grouting Material

**DOI:** 10.3390/ma19163396

**Published:** 2026-08-10

**Authors:** Yue Wu, Changwang Yan, Changan Miao, Junqing Li, Yanhui Li, Xiangdong Meng, Fengwei Zhao, Jie Liu

**Affiliations:** 1 School of Mechanics and Aeronautics, Inner Mongolia University of Technology, Hohhot 010051, China; 20201100310@imut.edu.cn; 2 School of Civil Engineering, Ordos Institute of Technology, Ordos 017010, China; 3 The Key Laboratory of Green Development for Mineral Resources, Inner Mongolia University of Technology, Hohhot 010051, China; 4 Inner Mongolia Autonomous Region Engineering Research Center for Eco-Building Materials and Fabricated Structure, Hohhot 010051, China; 5 School of Mining and Technology, Inner Mongolia University of Technology, Hohhot 010051, China; 6 School of Civil Engineering, Inner Mongolia University of Technology, Hohhot 010051, China; 7 School of Architectural Engineering, Hulunbuir University, Hulunbuir 021008, China; 8 Hulunbuir City Transportation Bureau Comprehensive Security Center, Hulunbuir 021008, China; 9 Huaneng Urad Middle Banner New Energy Power Generation Co., Ltd., Bayannur 015000, China; 10 College of Architecture, Inner Mongolia University of Technology, Hohhot 010051, China

**Keywords:** solid-waste binder, fluidity, silica fume, strength

## Abstract

With the continuous expansion of grouting material applications, solid waste to prepare grouting materials can effectively mitigate the environmental issues caused by waste accumulation. In this work, an all-solid-waste grouting material (ASWGM) was prepared with silica fume, coal gangue, desulphurization gypsum, fly ash, steel slag and carbide slag. The impact of water–cement ratio (W/C), environmental temperature, silica fume content, and hydration time on the fluidity of grouting materials was systematically analyzed. The chemical substances of such ASWGM were investigated by performing XRD and Fourier transform infrared spectroscopy measurements, while its fluidity was comprehensively evaluated by conducting apparent viscosity, yield stress, fluidity, and thixotropy tests. Based on our analysis, the optimal silica fume content was determined to be 5%, and 20 °C identified as the optimal environmental temperature. Under the preferred performing combination within the tested scope, the yield stress of the materials was 126.4 Pa. The incorporation of silica fume would decrease the early strength of the materials. The strength greatly increased in the late phase as the hydration continued. The strength exceeded 50 MPa at 12 h and 80 MPa at 28 d. Not only can the use of all-solid waste effectively solve the environmental pollution caused by solid waste stacking and facilitate green development, but also obtain grouting materials with satisfying fluidity and mechanical properties through design optimization. The research results can serve as a reference for guiding engineering practice.

## 1. Introduction

Cement-based grouting materials can be prepared by using various materials like aggregates as the supporting skeletal materials, cement materials like cement and fly ash as major binding agents, and mixing with additives (e.g., water reducer and expanding agent) [1]. Due to their comparative characteristics of early strength, high strength [2], no shrinkage [3], and microdilatancy [4], they have been extensively applied to various fields like architecture, mines and tunnels, accompanied by a continuous increase in demand [5]. Moreover, the foundation construction of high-rise buildings, underground parking lots, and railway stations imposes increasingly stringent requirements on the performance index of grouting materials. In the field of mining, grouting materials are used to fill and support the surrounding rocks, ensuring the safe operation of mines. This also places significantly higher demands on grouting materials. Nevertheless, traditional grouting materials mainly rely on the non-renewable resource of cement, which consumes significant energy and generates substantial carbon emissions during their production process. Developing grouting materials from solid wastes and advancing their applications represent a critical technological direction [6]. At present, stacking of traditional industrial solid wastes (e.g., desulphurization gypsum, fly ash, coal gangue, silica fume, and carbide slag) and improper processing are causing environmental damage and resource waste. To date, the technology for using slag, carbide slag, coal gangue and metakaolin to enhance the performance of cement-based materials is relatively mature [7,8,9]. Cement prepared from all-solid-waste materials exhibits early high-strength mechanical properties and environmental benefits due to its low calcination temperature [10,11]. Recently, significant advances have been made in developing sustainable grouting materials and alkali-activated binders incorporating various industrial solid wastes, demonstrating great potential in reducing carbon footprint and optimizing rheological performance [12,13]. Therefore, the production of grouting materials by making full use of solid waste resources has become a key technological approach for achieving sustainable development of the architectural industry.

The impact of solid wastes (e.g., silica fume, fly ash, and coal gangue) on the fluidity of ordinary grouting materials has been extensively investigated in the literature. The addition of fly ash, coal gangue, and silica fume mixtures into ordinary cement-based grouting materials could increase the generation of ettringite in the slurry in the late phase, offsetting the late structural shrinkage of grouting materials [14,15]. In the dual-mixture system of fly ash and coal gangue, it has been found that fluidity reaches the peak at a low content of the mixture. When the fly ash content slightly exceeds that of the coal gangue content, its contribution to the sample’s strength is more pronounced [16]. In the single-mixture system, it has been found that the incorporation of an appropriate amount of fly ash will not only significantly increase fluidity, but also accelerate the consumption of cement clinker and increase the hydration rate. The late strength is substantially greater than that of the ordinary Portland cement, promoting microstructural refinement and thereby improving the durability of the material [17,18]. Additionally, it has been noted that under the collaborative effect of fly ash and coal gangue, fly ash can accelerate the pozzolanic reaction, which is necessary to form tobermorite (C-S-H) gel [17]. Therefore, fly ash is widely regarded as an essential component in grouting material mixtures. Silica fume, another highly effective material, offers high reactivity and extremely fine particle size, enhancing grout fluidity [19]. The major chemical component of silica fume is SiO_2_, which reacts with cement-derived Ca(OH)_2_ to form C-S-H gel. The latter can fill the gaps in the concrete and thereby enhancing the compactness of the concrete structure [20]. Silica fume can also optimize the fluidity of different cements to different extents. According to the literature, silica fume can improve the fluidity of Portland cement better than other cements [21,22]. Meanwhile, recent studies on silica fume application have highlighted its synergistic microstructural packing and pozzolanic effects in multi-component cementitious systems [23,24]. However, the dynamic rheological evolution, thixotropic behavior, and structural build-up of all-solid-waste grouting materials without traditional Portland cement remain to be fully elucidated, particularly under varying thermal and operational conditions.

Additionally, the fluidity of grouting materials can often be significantly improved by optimizing the grading and types of fine aggregate. At present, river sand, standard sand, and quartz sand [25,26] are the major aggregates; steel slag, an industrial waste produced by steel enterprises in large quantities, has not been effectively used. Stacking or landfilling steel slag consumes valuable land and poses environmental pollution risks. Steel slag is porous and has high strength. It is often used as large-scale substitution of aggregates in concrete [27,28]. Moreover, it has good mechanical properties. To this end, the utilization of fine-grained steel slag as aggregates of solid-waste grouting materials can lower the proportion of cementing materials and reduce energy consumption during the preparation process. Moreover, the high porosity can slowly release cementing materials and increase their fluidity [29]. W/C is the most intuitive way to increase the fluidity. The selection of an appropriate W/C that will yield good mechanical properties [30,31] is the key point to determine the mix proportion of the materials. In addition, both the environmental temperature [32,33] during construction and the control of the hydration time [34] can affect the fluidity of grouting materials. These factors should be meticulously considered during the transportation and construction of such materials.

Although various solid-waste-based grouts have been reported, most existing studies still rely on partial Portland cement clinker as an activator or focus primarily on single-parameter mechanical performance, leaving a knowledge gap regarding the dynamic rheological behavior and structural build-up of ASWGM. Furthermore, the multi-phase synergistic mechanism of silica fume within a cement-free matrix under varying thermal environments and hydration intervals remains insufficiently understood. Along these lines, in this work, all-solid-waste cement was prepared through carbide slag, coal gangue, and desulphurization gypsum. Moreover, the fine-grained steel slag was used as the aggregate, while fly ash and silica fume were used as fillers to prepare grouting materials. The primary novelties and scientific contributions of this work are threefold:

(1) Energy conservation and environment protection: This approach not only yielded a significantly lower consumption of traditional cement, but also permitted the effective resource recycling and reduced the stacking of industrial solid wastes, as well as environmental pollution.
(2) Rheological and thixotropic mechanism: Elucidating the dynamic rheological evolution, yield stress response, and quantitative thixotropic recovery of the cement-free slurry modulated by silica fume micro-filling and pozzolanic effects.
(3) Environmental and temporal sensitivity: Quantifying the sensitivity of viscosity and structural rebuilding to environmental temperatures (10–25 °C) and hydration times; establishing rational operational windows for practical grouting applications.

## 2. Materials and Methods

### 2.1. Materials

**Figure 1:**
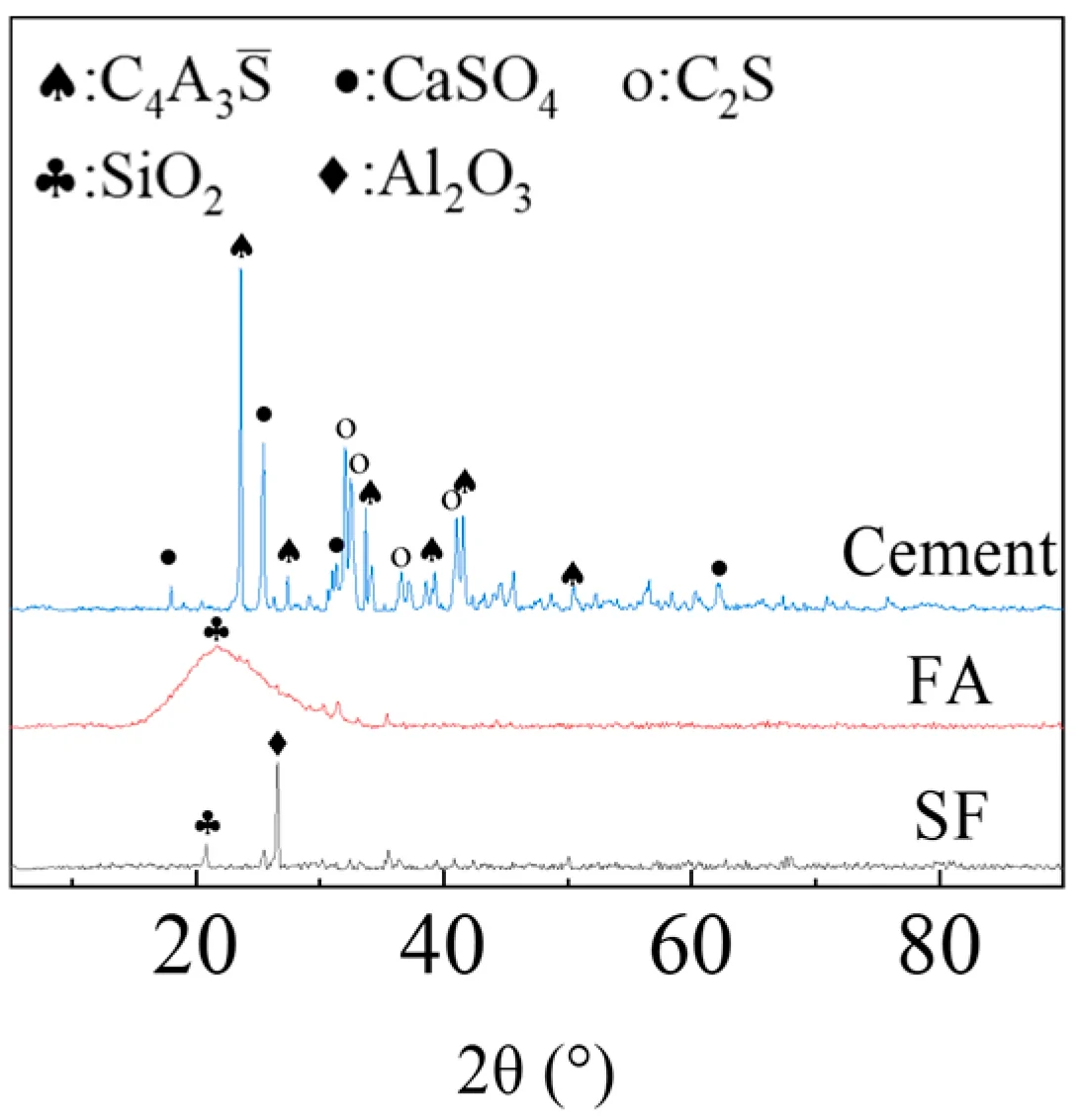
XRD composition of raw materials.

The working-cementing materials were prepared by coal-based solid waste, and the chemical components of coal gangue(Ordos, China), carbide slag(Ulanqab, China), and desulphurization gypsum(Shijiazhuang City, China) are presented in Table 1. Specifically, the aforementioned solid waste materials were first screened by a 200-mesh sieve(Zhejiang Shangyu Shengchao Instrument & Equipment Co., Ltd., Shaoxing, China). Second, the processed coal gangue, desulphurization gypsum, and carbide slag were mixed according to predetermined ratios [35], with 20% test water added. Third, the raw materials were compressed into a Φ60 × 6 mm cylindrical specimen by a tablet machine(FTLD24Y-30, Henan Shangqiu Futong Machinery Equipment Co., Ltd., Shangqiu, China). Fourth, the specimen was put in furnace(KSL-1700X-A4, Hefei Kejing Material Technology Co., Ltd., Hefei, China) for insulated calcination according to the calcination scheme determined by previous studies [7], thus obtaining the solid waste cementing material. Finally, the solid waste cementing material and gypsum were mixed at a ratio of 100:3 and then ground with a cement test grinding machine(TM-400, Beijing Minghong Weiye Technology Development Co., Ltd., Beijing, China). The cementing material for testing was obtained by sieving the powder through a 200-mesh screen, achieving a residue content of less than 3%. The XRD spectrum of the prepared solid waste cementing material is presented in Figure 1.

The I-level fly ash(Ordos, China) was used as the mineral additive. The density, specific surface area, water demand, and 28d activity index of the fly ash were 2.25 g/cm^3^, 373 m^2^/kg, 92% and 73%, respectively. All indicators of fly ash conform to the requirements of I-level fly ash in GBT1596-2005 Fly Ash for Cement and Concrete [36]. The chemical composition and the XRD spectrum of the fly ash are respectively listed in Table 1 and shown in Figure 1.

The S95-level silica fume(Zhengzhou City, China) was used as the mineral additive. The density, specific surface area, water demand, and 28d activity index of the silica fume were 2.26 g/cm^3^, 12,182 m^2^/kg, 121% and 92%, respectively. All indicators of silica fume conform to the requirements in GBT27690-2011 Silica Fume for Mortar and Concrete [37]. The chemical composition of silica fume is listed in Table 1 and XRD spectrum of silica fume is shown in Figure 1.

The steel slag sand(Baotou City) served as the fine aggregate. Impurities in the steel slag sand were eliminated and then the steel slag sand was cleaned with tap water. The cleaned steel slag sand was dried indoors. The performance indicators of steel slag sand are listed in Table 2. The fractal dimension of aggregate ranges between 2.4 and 2.8. The fineness modulus was 3.1~3.7 and the average grain size was over 0.5 mm.

The high-performance polycarboxylate water reducer(Chengdu City, China) was applied, which has good dispersibility, water-reducing performance, and high dissolution in water. In order to effectively prolong the setting time of cement, tartaric acid(Chengdu City, China) was used as the retarder. In this work, the mass ratio of additives and the total mass of cementing materials, fly ash, and silica fume was 0.0065:1. The ratio of water reducer and retarder was 10:3. The ordinary tap water was applied as the mixing water.

### 2.2. Preparation of ASWGM

(1) Fly ash and silica fume were used as mineral additives. The mixing ratio of different materials was determined according to their specific surface areas, grain size distribution, and chemical components.
(2) Raw materials were weighted. Additives were added into weighted water and stirred. Later, the mixing uniformity was controlled according to the preset stirring time.
(3) Key properties of the stirred slurry were tested. According to the requirements of GB/T50448-2015 [38] standard, the mixing ratio of raw materials was adjusted, finally determining the basic mixing ratio for the test. More details are shown in Table 3. Basic physical properties like initial setting time, final setting time, vertical expansion rate, initial fluidity, fluidity at 30 min and 28d compressive strength are listed in Table 4.

### 2.3. Test Methods

#### 2.3.1. Rheological Experimental Test

The HAAKE™ Viscotester™ iQ rheometer of Thermo Fisher Scientific Inc. (Waltham, MA, USA) was used. The viscosity and shear stress were calculated and the curves were plotted in the data analysis software. The applied combination was a blade rotor (model: FL16 4B/SS–01210364) and a coaxial cylinder. To eliminate the potential influence of other variables, the test time was set prior to the initial setting time. The mixing protocol for grouting materials was consistent across all rheological tests.

**Figure 2:**
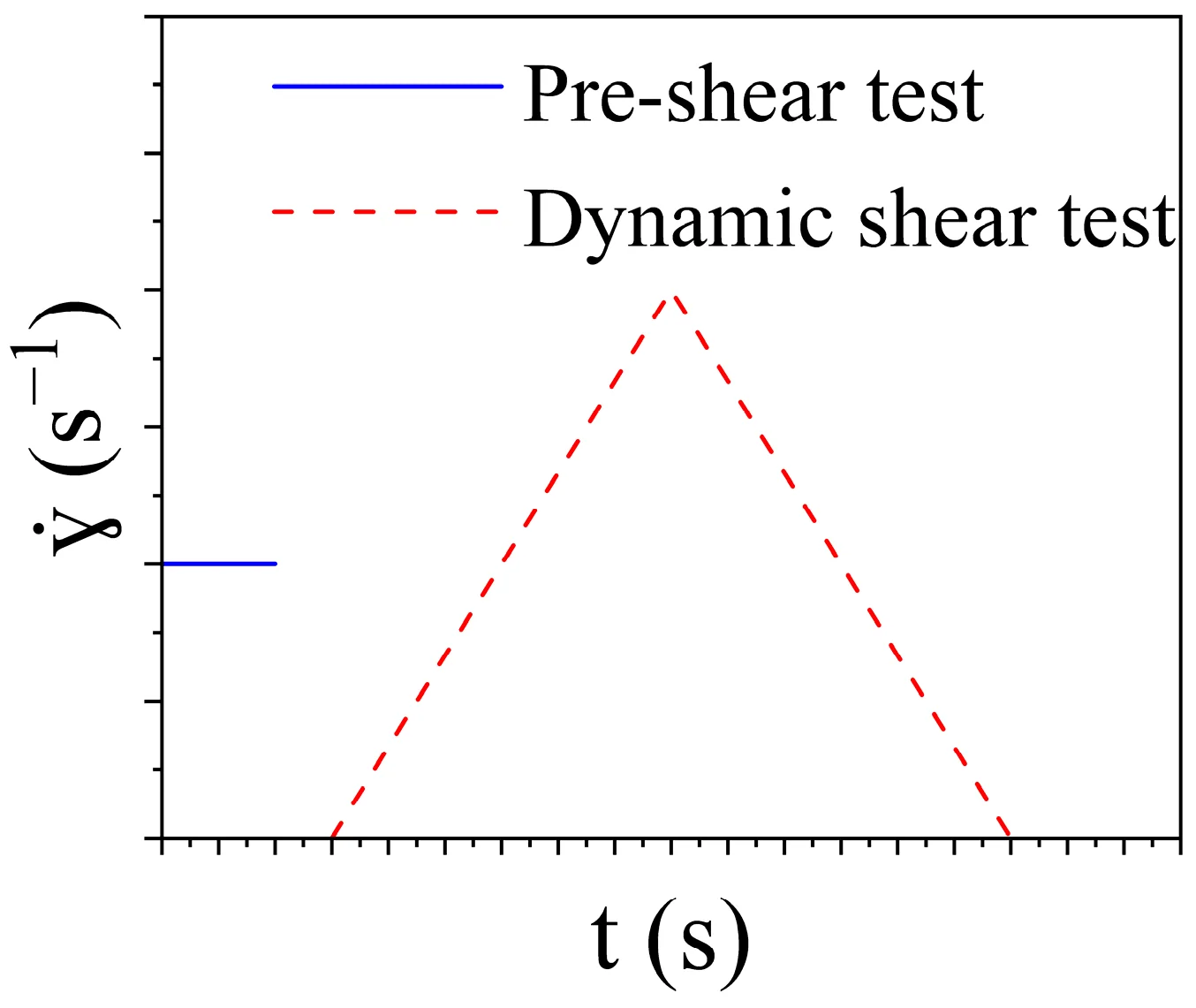
Shear scheme of the rheological test.

The test stages of grouting materials are shown in Figure 2. The pre-shear stage at 50 s^−1^ constituted the initial step. The goal of this stage is to disperse the slurry and ensure that it has some initial state before the dynamic shear stage. Following the initial pre-shear stage at a constant 50 s^−1^, the dynamic shear stage commenced. During this second stage, the shear rate ramped linearly from 0 to 100 s^−1^ and back down to 0. Data from the descending shear rate ramp was analyzed to determine variations in yield stress and viscosity across different shear rates. This analysis yielded the relationship curves showing how yield stress and viscosity depend on shear rate.

**Figure 3:**
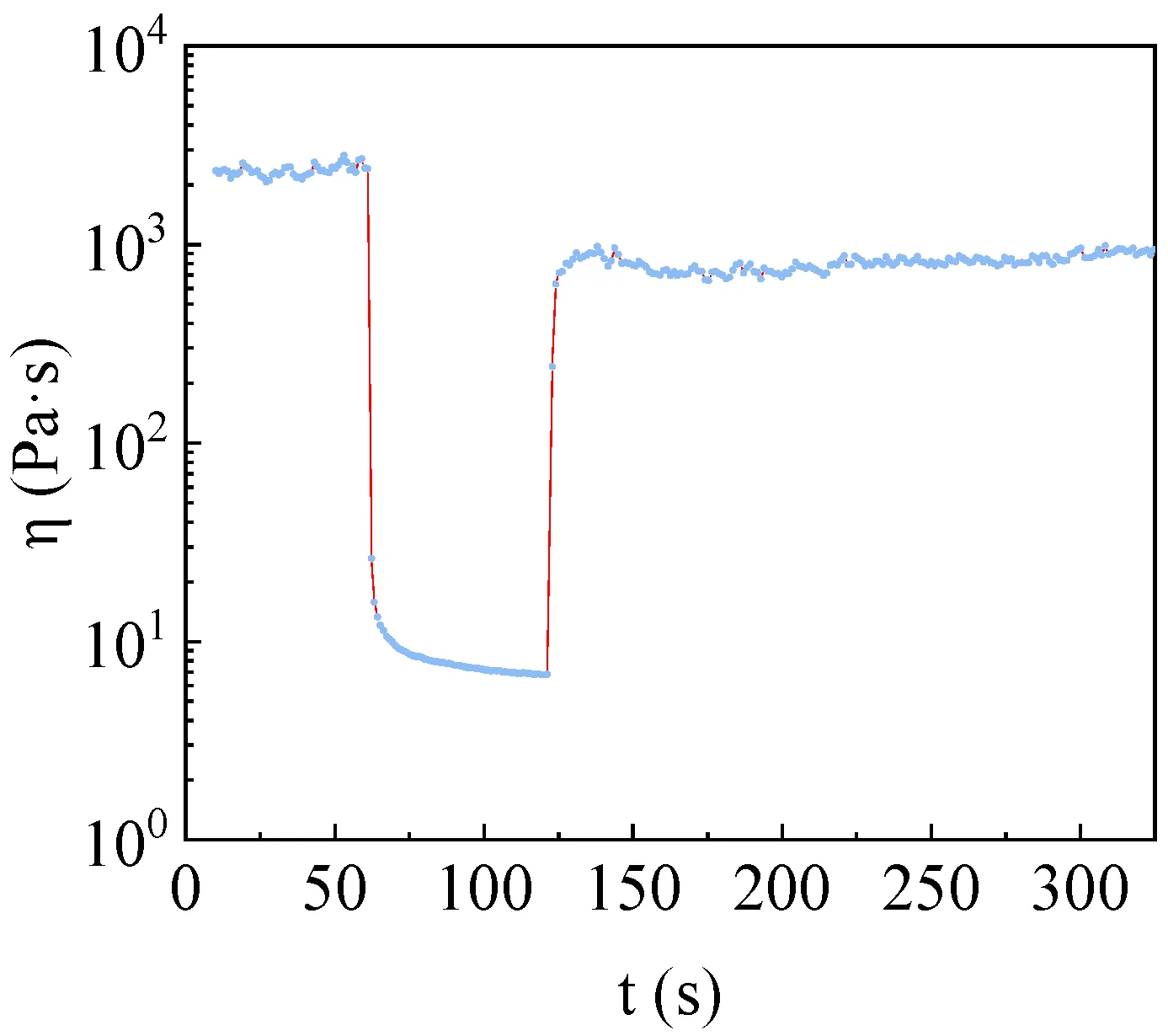
Thixotropy rheological model.

The materials presented shear thinning in the test. The rheological curve, which was fitted by test results, showed strong correlations with the fluid equation of the Herschel–Bulkley model (H–B model) [39]. Moreover, the rheological models at different moments all conformed to the H–B model, with R^2^ higher than 0.97. The fitting results showed high reliability. Figure 3 presents the rheological curves obtained for the ASWGM. In this work, the H–B fluid model was applied for fitting of rheological behaviors of the grouting materials.

#### 2.3.2. Microscopic Testing

Phase analysis: The microstructures of raw materials for preparing grouting materials, slurry samples and hardened samples were analyzed by performing XRD measurements(Ultima IV, Rigaku Corporation, Tokyo, Japan). The scanning rate and scanning angle were 0.02°/s and 5–90°, respectively. Phase analysis of test results was carried out by using Jade data analysis software (Version 6.5, Materials Data, Inc., Livermore, CA, USA).

Evolution of functional groups: Fourier transform infrared (FTIR, IRTracer-100, Shimadzu Corporation, Kyoto, Japan) spectroscopy analyzed the functional groups of hydration products in grouting materials at different hydration stages, using a scanning range of 440–4700 cm^−1^. In the experimental process, the background was scanned every certain period to ensure the test results.

#### 2.3.3. Macro Testing

Compressive strength: Grouting materials were cast into triplicate cubic specimens (40 mm × 40 mm × 40 mm), which were then cured under standard conditions for a predetermined period. Compressive strength was tested using a universal servo testing machine(WDW-100E, Jinan Fangyuan Testing Machine Co., Ltd., Jinan, China) at a loading rate of 2400 N/s.

Fluidity: It was tested according to JGT408-2019 *Sleeve Grouting Materials for Connection of Steel Bar* [40]. The frustum model (upper mouth: 70 mm; lower mouth: 100 mm; height: 60 mm) was applied. The glass plate was secured on the horizontal plane during the test. After initial thorough stirring, the slurry was poured into the frustum model, which was then lifted vertically to allow the pure slurry to flow onto the glass plate for 30 s. The mean of the maximum diffusion diameter at the bottom test surface and the diameter perpendicular to the bottom test surface was chosen as the final fluidity.

Grading analysis: The cementing material and aqueous solution were mixed according to the preset ratio. The stirred slurry was put in the curing chamber until the preset time. The mixture was then put in a beaker and stirred fully with a piece of glass rod. An appropriate amount of slurry was collected by a dropper and then added into a beaker with 40 mL absolute ethyl alcohol. The mixture was stirred fully again. Later, the grain size distribution of raw materials was tested by the LS-POP9 laser particle size analyzer (Zhuhai Aomeike Instrument Co., Ltd., Zhuhai, China). Absolute ethyl alcohol was chosen as the dispersion medium and the rotation speed of the stirrer was set at 2500 r/min.

All rheological measurements were performed under temperature-controlled laboratory conditions (20 ± 2 °C). Each rheological protocol was repeated at least twice to ensure high reproducibility (RSD < 5%). For compressive strength testing, three identical specimens (N = 3) were tested for each mix formulation and curing interval, with mean values and standard deviations reported. The W/B ratio was selected to meet the dual criteria of field pumpability (fluidity >30 cm) and high early strength.

## 3. Results and Discussion

### 3.1. Impact of Silica Fume Content on the Rheological Properties of ASWGM

**Figure 4:**
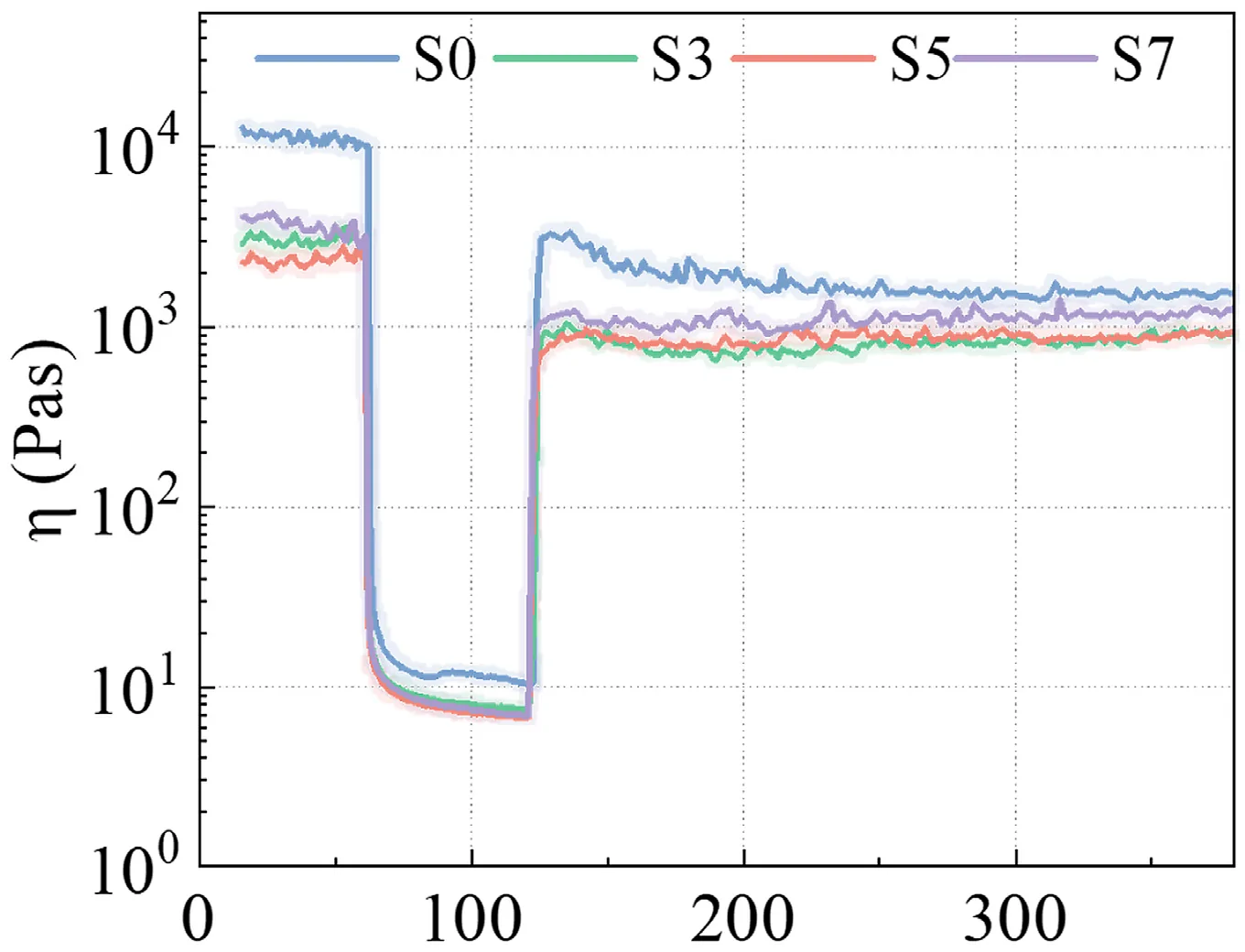
Thixotropy of ASWGM with different silica fume contents.

**Figure 5:**
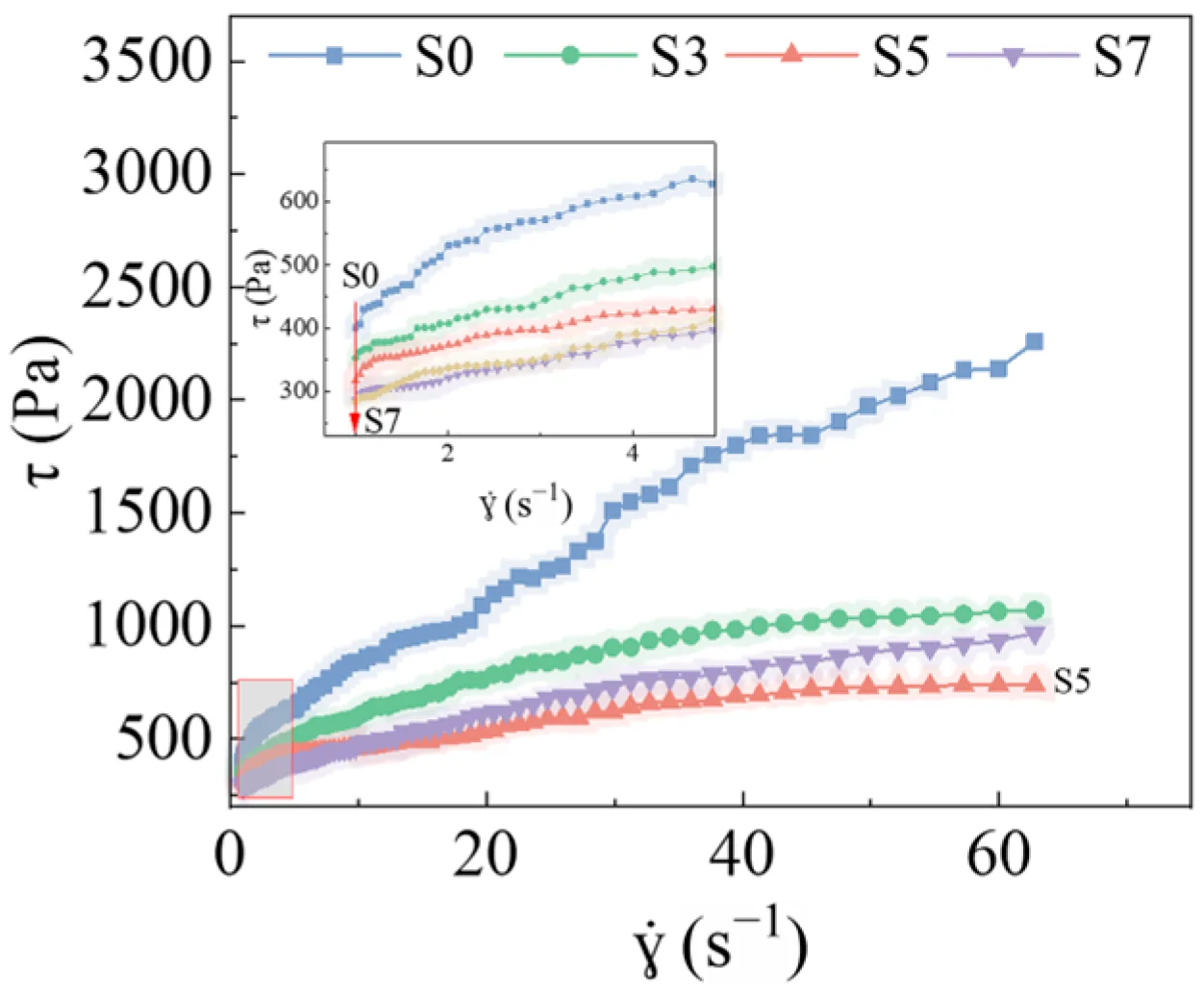
Shear stress of ASWGM with different silica fume contents.

The thixotropy test results are shown in Figure 4. S0, S3, S5 and S7 were used to express the thixotropic recovery property of the grouting materials at 60.00 s after the shearing rate changes, with values of 20.54%, 26.10%, 27.10%, and 30.28%, respectively. With the increase in the silica fume, the thixotropic recovery property gradually increased, reaching the peak at S7. It can also be seen from Figure 5 that under a relatively high shearing rate, the state changed from dynamic to static. The absolute variations in thixotropy of S0, S3, S5, and S7 at the end of structural recovery were 8558 Pa·s, 2100 Pa·s, 1418 Pa·s, and 1953 Pa·s respectively. Obviously, the absolute variation in thixotropy of the grouting materials with silica fume significantly decreased, reaching the valley at S5. This result indicates that S5 has good fluidity, because silica fume particles in the system can promote recovery of the static state after flowing. Accelerated restoration of the internal network structure leads to increased thixotropy in the slurry, which is beneficial for internal-forming post-casting. Moreover, SiO_2_ and Al_2_O_3_ are major components of silica fume, which react with alkaline substances produced during hydration in the slurry, thus lowering the alkali content of the grouting materials while enhancing their compactness and fluidity. However, excessive silica fume content can lead to intensified reactions that may adversely affect the optimization of fluidity.

Figure 5 illustrates the rheological curves under varying silica fume contents, specifically depicting the relationship between shear rate and shear stress. The rheological curves were fitted using the Herschel–Bulkley model (${\tau =\tau }_{0}+K{\dot{\gamma }}^{n}$) with high correlation coefficients (R^2^ > 0.98). Across all silica fume dosages, the flow behavior indices (*n*) were strictly below 1.0 (ranging from 0.72 to 0.88), quantitatively confirming that the all-solid-waste slurry exhibits typical shear-thinning (pseudoplastic) behavior rather than dilatant flow. Specifically, as the silica fume content increased from 0% to 5%, the yield stress (τ_0_) and consistency index (K) dropped from 215.3 Pa and 18.6 Pa·sn to 126.4 Pa and 9.4 Pa·sn, respectively. This demonstrates that ultra-fine silica fume liberates trapped pore water and provides a ‘ball-bearing’ lubrication effect, significantly enhancing slurry flowability while maintaining pseudoplastic character.

At the beginning of loading, the shear stress gradually declined with the increase in the silica fume content manifested as S7< S5 < S3 < S0. This result demonstrates that given a low shearing rate, the rheological properties can be significantly optimized by increasing the silica fume content because it has a regular surface. The surface with greater roundness can develop the ‘ball lubrication effect’ and increase the fluidity. Nevertheless, the shear stress of S7 became gradually higher than that of S5 with the increase in the shearing rate, specifically, S5 < S7 < S3 < S0. This outcome reflects that S5 has better rheological properties. This behavior originates from the changing overall grain size distribution of grouting materials under different silica fume contents and the silica fume that can fill in gaps fully under an appropriate content. Therefore, the overall grading can be optimized and the bulk density of cementing materials can be increased, yielding an enhanced fluidity.

**Figure 6:**
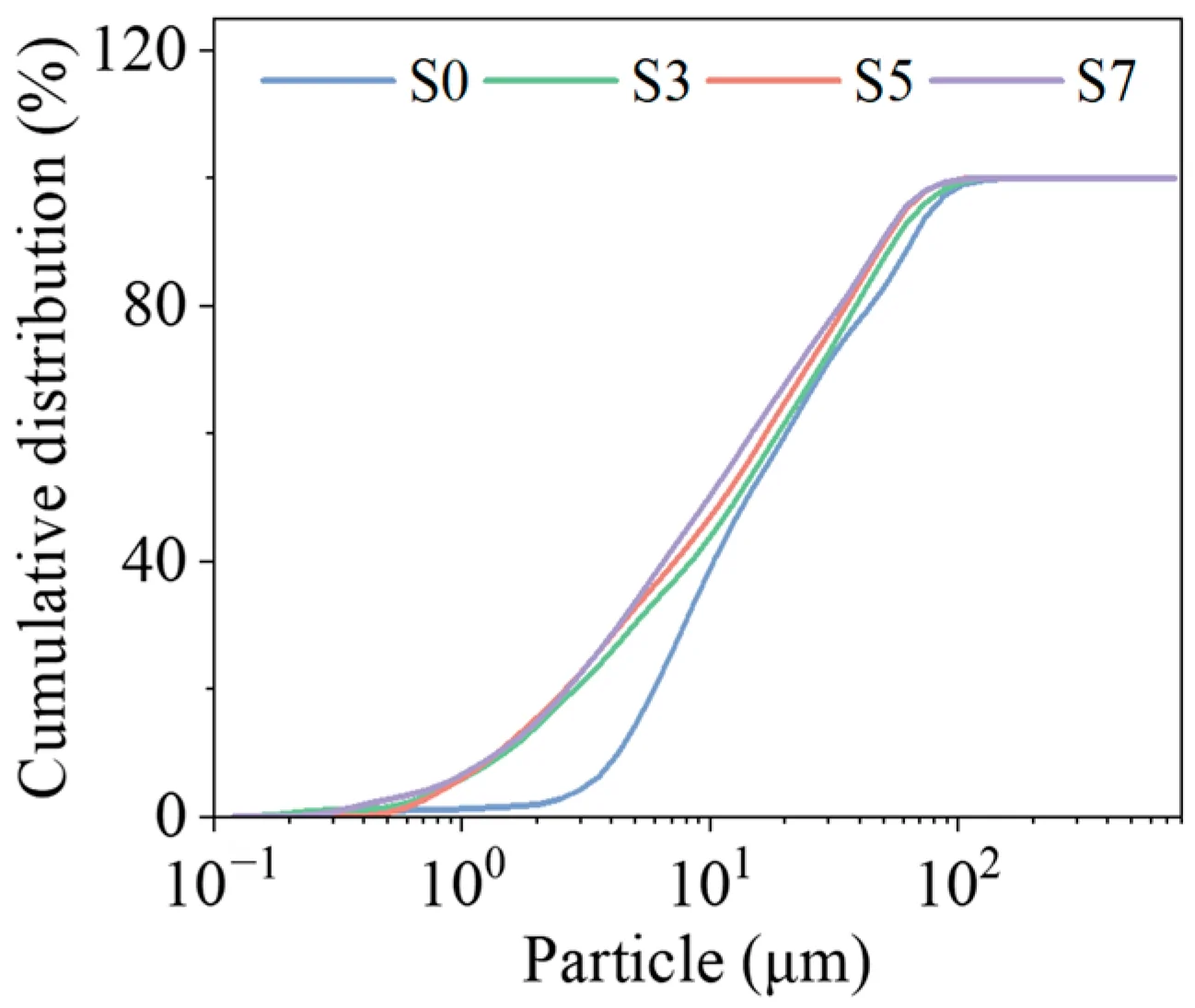
Grain size distribution of ASWGM with different silica fume contents.

The grain size distribution under different silica fume contents is shown in Figure 6. With the increase in the silica fume content, the grain size distribution (<10 μm) of the grouting materials was optimized and the curve becomes fuller. At S5, it was close to the optimal grading curve (S-shaped). This effect further demonstrates that silica fume has remarkably high specific surface area since its grain size is far lower than that of other mineral additives. With increasing silica fume content, the rheological properties of cementing materials with mineral additives can be significantly affected. The better grading curve reflects the enhanced fluidity.

### 3.2. Impact of Environmental Temperature on the Rheological Properties of ASWGM

As one of the most important influencing factors of the hydration process, temperature is crucial to fluidity. Establishing the appropriate environmental temperature serves as an important parameter for the transportation and mixing of grouting materials. As can be seen, S5 exhibits good fluidity and grading distribution. S5 was chosen as the research group to investigate the influence of environmental temperature on the slurry’s rheological properties. The rheological properties of the slurry in the temperature range of 10~25 °C were thoroughly investigated.

**Figure 7:**
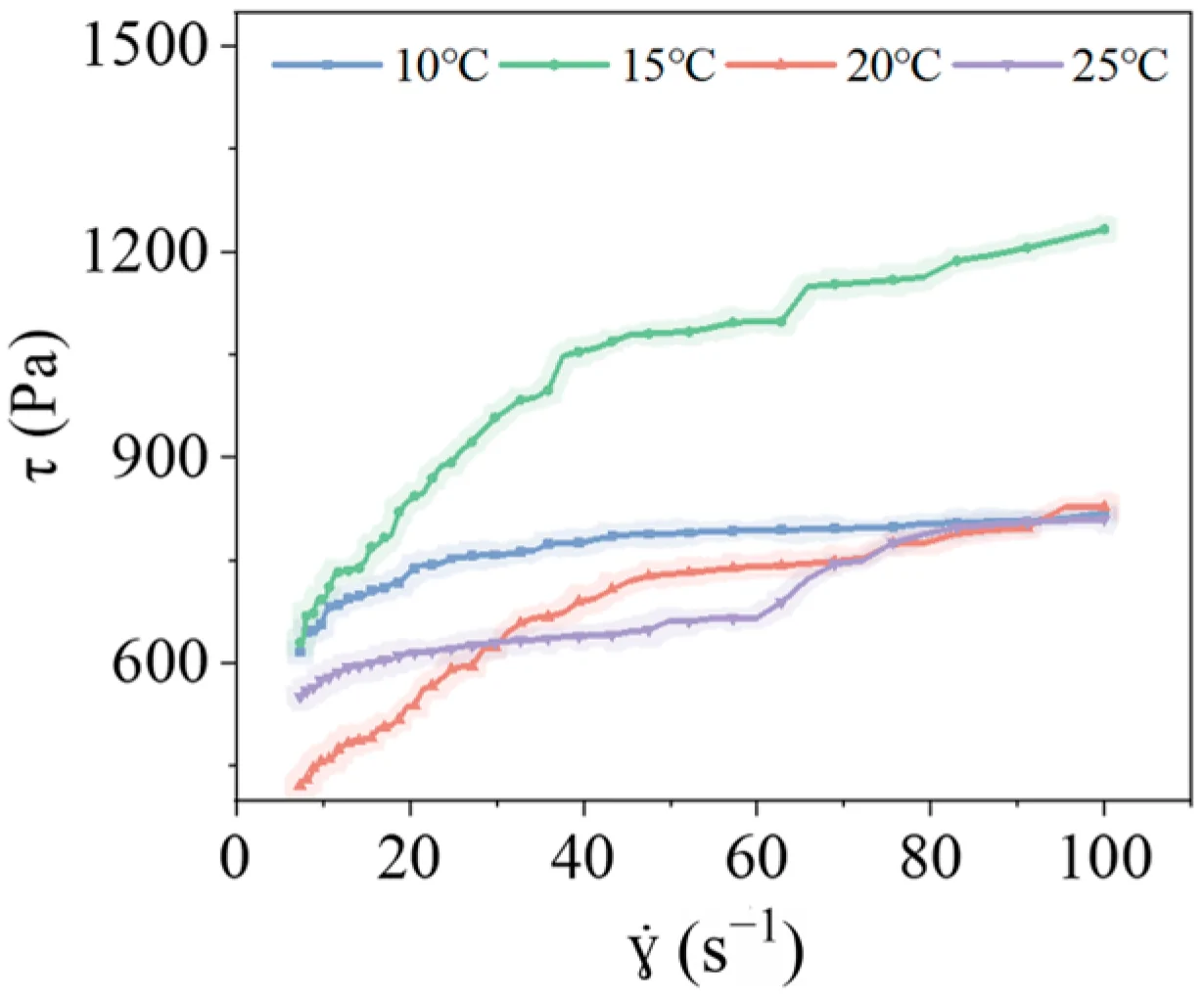
Shear stress of ASWGM under different environmental temperatures.

The rheological curves of ASWGM under different temperatures are shown in Figure 7. As can be seen, in the beginning of shearing, the shear stress was relatively low at 20 °C. As the shearing rate increased, the shear stress of various temperature groups gradually increased. However, the growth trend showed different variation states due to the influence of the environmental temperature. Specifically, the shear stresses of the 15 °C and 20 °C groups were significantly affected when the shearing rate was 20^−1^–40 s^−1^, while the shear stresses of the 10 °C and 25 °C groups slowly changed. In the final stage, the shear stress of the 15 °C group was far higher than that of the other three groups.

**Figure 8:**
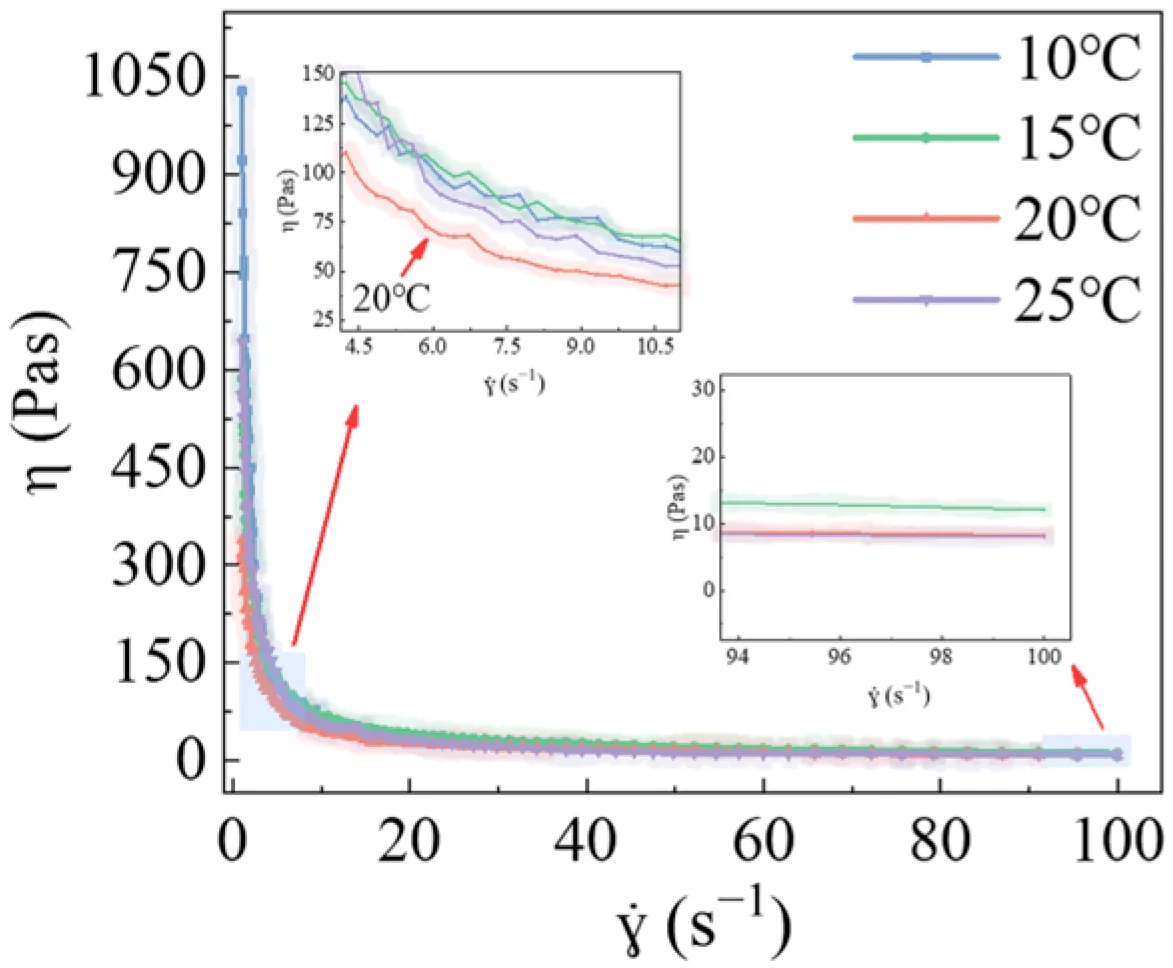
Apparent viscosity of ASWGM under different environmental temperatures.

The variations in the apparent viscosity of the grouting materials with temperature are shown in Figure 8. According to the rheological curves, the slurry viscosity was low given the fixed shearing rate, thus decreasing the retardation to the rotor. The apparent viscosity decreased with increasing shearing rate, and the slurry exhibited obvious shear-thinning behavior. From the rheological curve, it can be inferred that the apparent viscosity also showed a gradual decrease with increasing temperature. The 20 °C group showed good fluidity. The apparent viscosity curves of the 10 °C and 20 °C groups were compared. Based on our analysis, it was revealed that the temperature influences the viscosity of the section with low shearing rate (0–20 s^−1^) and the viscosity coefficient decreased to 53.25% of its original value. The slurry showed strong fluidity and the compactness of the particles increased. However, the viscosity gradually increased as the temperature elevated from 20 °C to 25 °C. This is because the hydration induction period was shortened by increasing the environment temperature, while the hydration heat-releasing rates in the induction period and acceleration period were increased. The internal energy of the grouting material-slurry gradually increased, thereby accelerating the thermal motion of molecules and particles. The water evaporated and the adhesion effect of particles was accordingly weakened [41].

**Figure 9:**
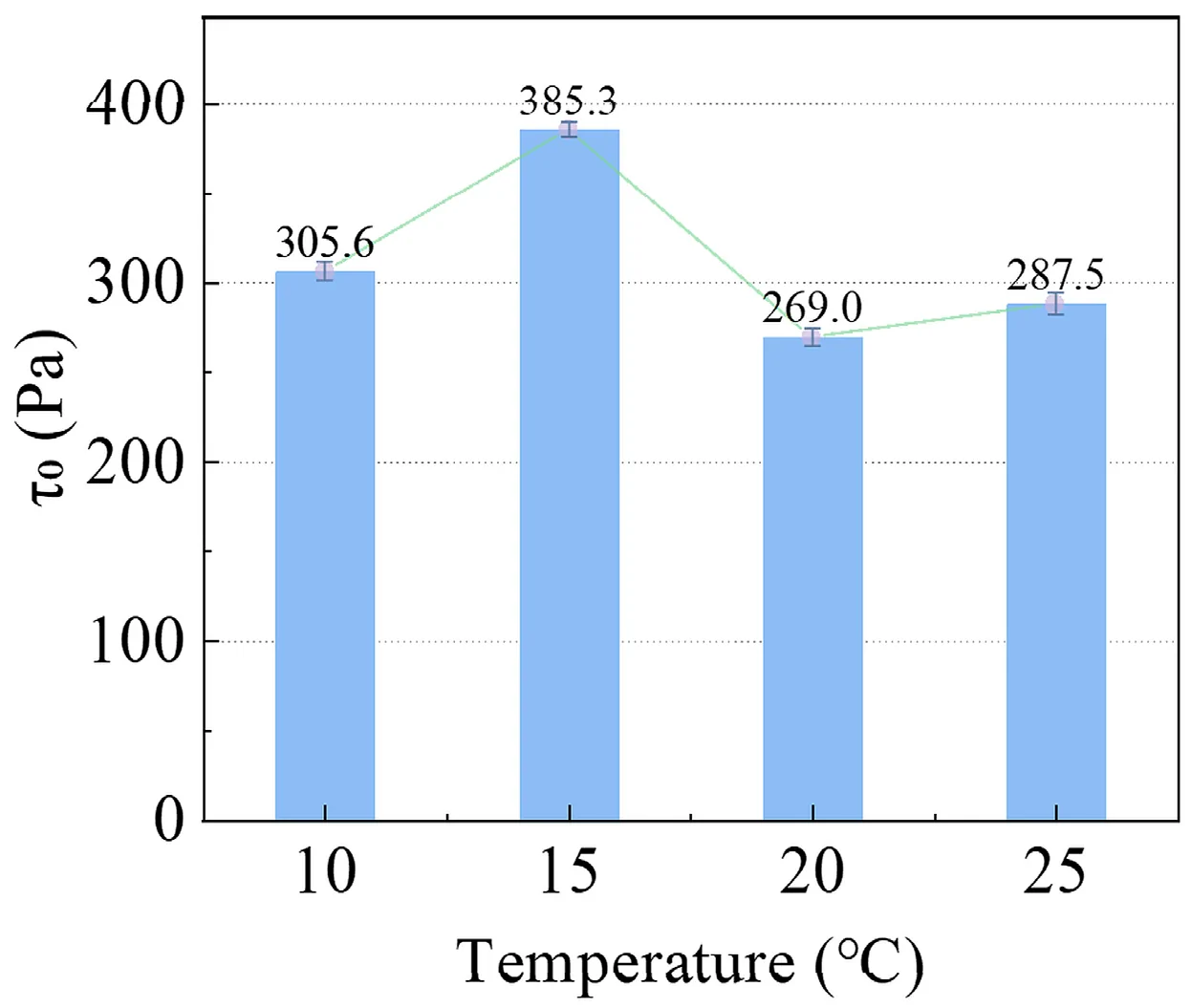
Yield stress of ASWGM under different environmental temperatures.

The yield stress fitted from the H–B fluid model is shown in Figure 9. Obviously, the yield stress of the slurry changed within the range of 250~300 Pa when the temperature increased from 10 °C to 25 °C. The variation range of stress was within 100 Pa. The shear stress of the slurry reached its maximum at 15 °C and its minimum at 20 °C, accompanied by the lowest flow resistance. This finding aligns with the variation patterns of apparent viscosity and shear stress. Therefore, 20 °C was determined as the optimal environmental temperature.

### 3.3. Impact of the W/C on the Rheological Properties of Fresh Grouting Materials

**Figure 10:**
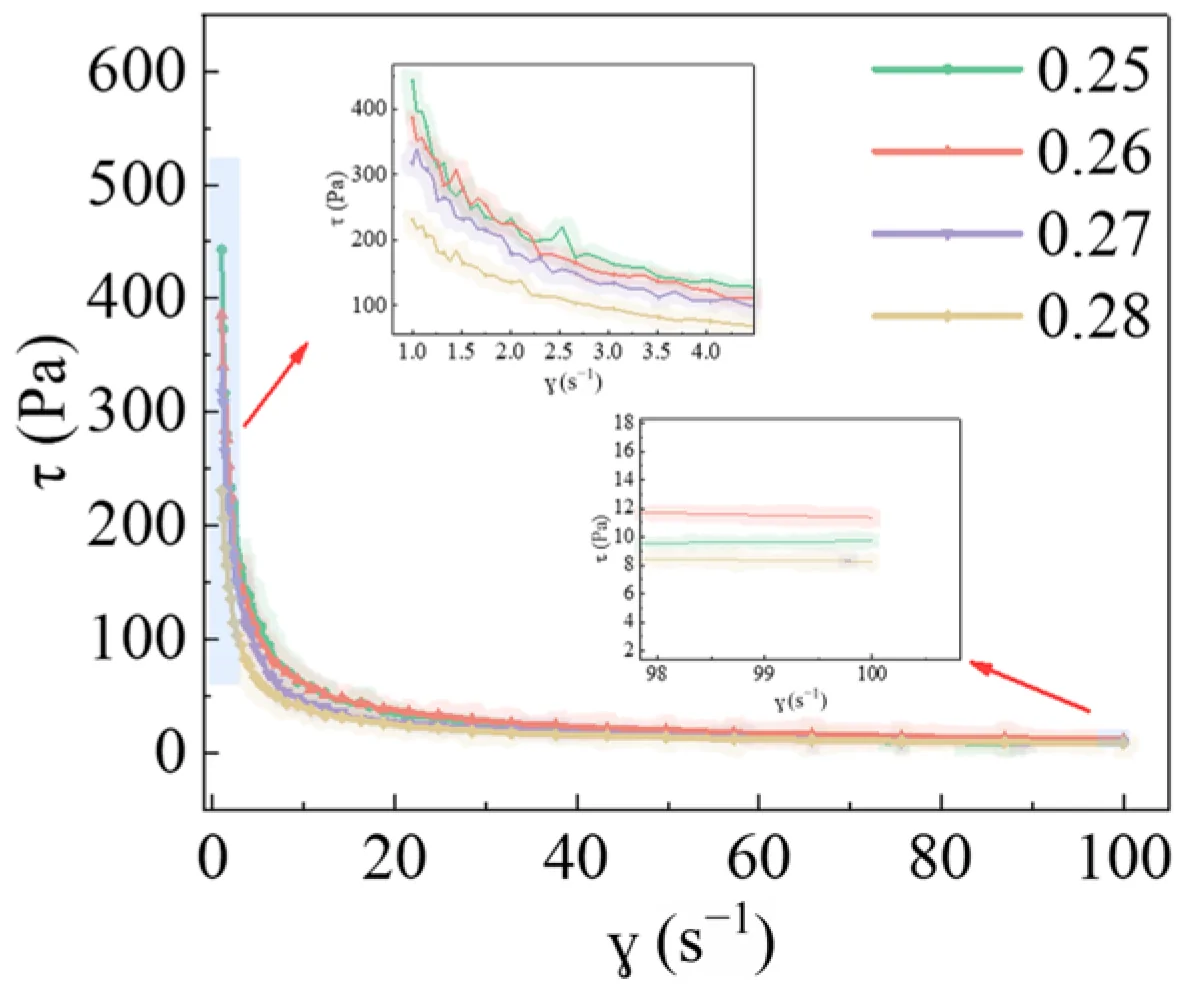
Apparent viscosity of ASWGM under different W/C.

**Figure 11:**
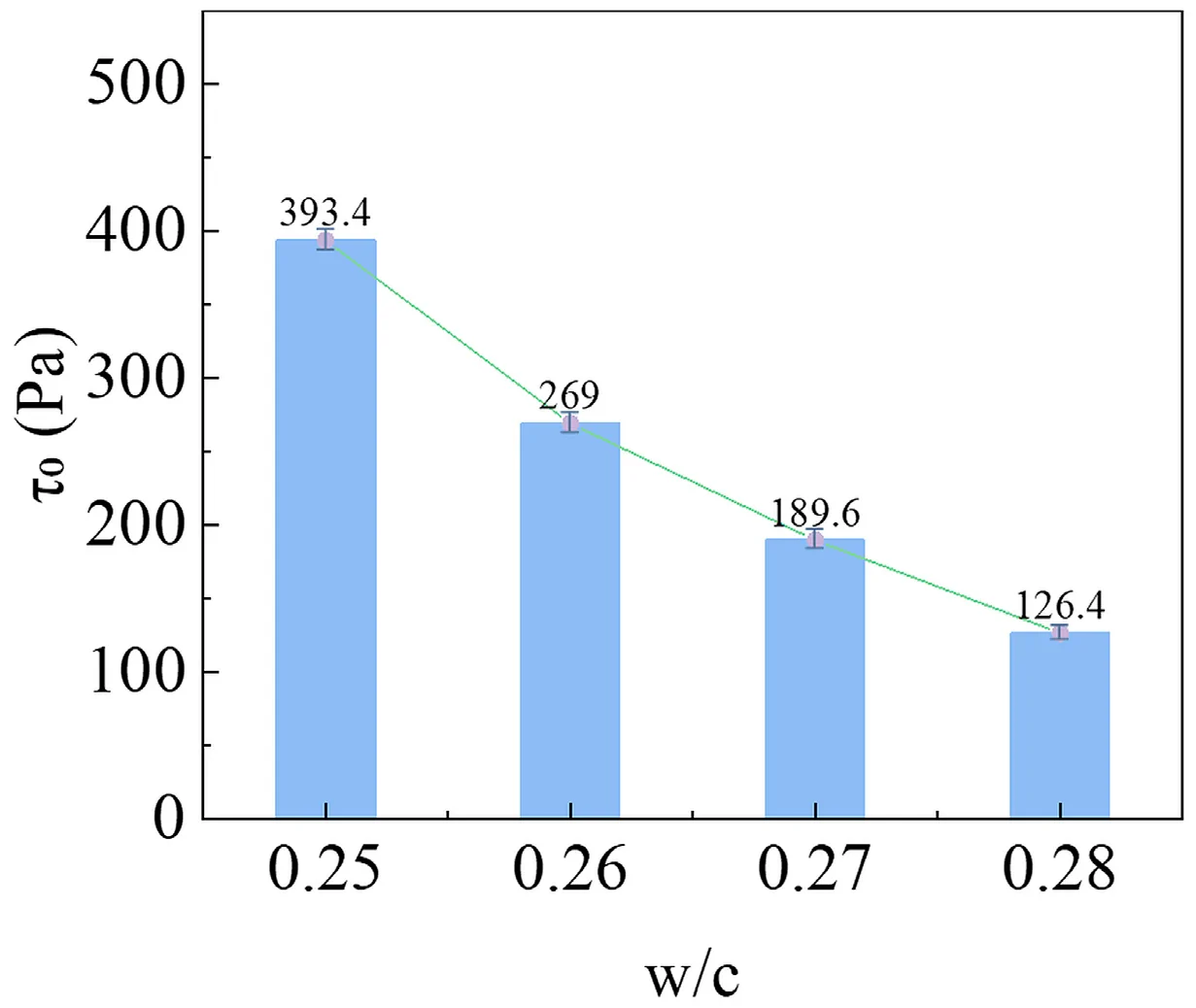
Yield stress of ASWGM under different W/C.

Adjusting the W/C can effectively improve the flow performance of grouting material. Figure 10 presents the variation trend of apparent viscosity of grouting material under different W/C. It can be observed that as the W/C increases, the viscosity decreases in the low shear rate range (0–20 s^−1^). However, the former were almost consistent in the section with high shearing rates (80–20 s^−1^). Later, the yield stresses under different W/C, which were calculated from the H–B model, were analyzed (Figure 11). With the increase in the W/C, the yield stress gradually decreased. Particularly, a 0.01 increase in the average W/C caused yield stress to decrease by 84.725 Pa. In addition, the W/C exerts a considerably greater influence on the rheological parameters than other factors.

**Figure 12:**
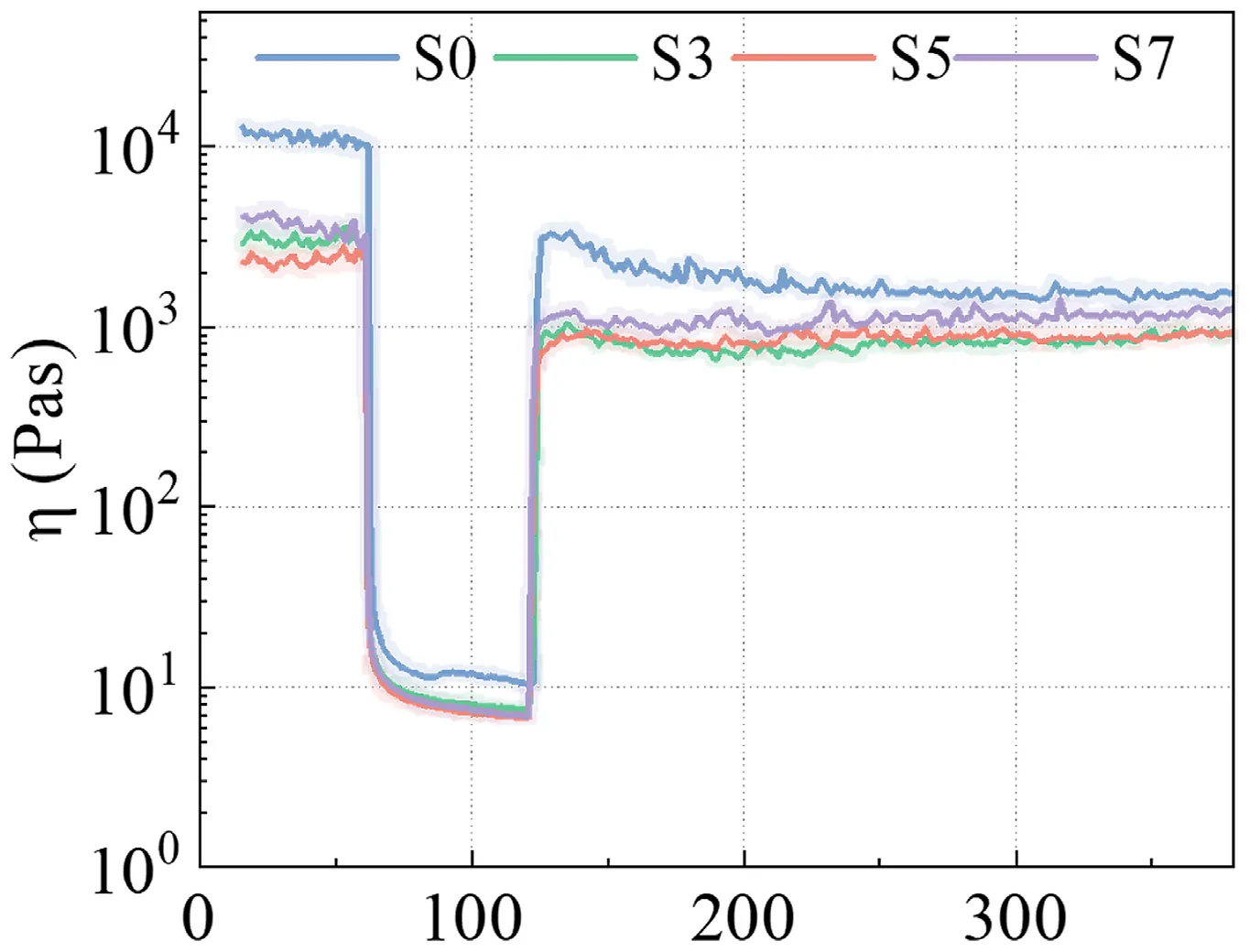
Thixotropy of ASWGM under different W/C.

The test results of the thixotropy of grouting materials under different W/C are shown in Figure 12. It can be seen that the increase in W/C can cause the grouting slurry in a static state to aggregate rapidly. The absolute variations in the slurry with W/C of 0.25, 0.26, 0.27, and 0.28 at the end of recovery were 6579 Pa·s, 1418 Pa·s, 899.5 Pa·s, and 254.3 Pa·s, respectively. The viscosity under different W/C of 0.25, 0.26, 0.27, and 0.28 was recovered to 16.28%, 27.10%, 35.61%, and 51.25% of the beginning value (60.00 s). The viscosity recovery efficiency relative to the beginning of high shearing rate was positively related to the W/C. This outcome reflects that the thixotropy of the ASWGM can be improved by adjusting water consumption. Hence, slurry can adapt to complicated construction conditions.

**Figure 13:**
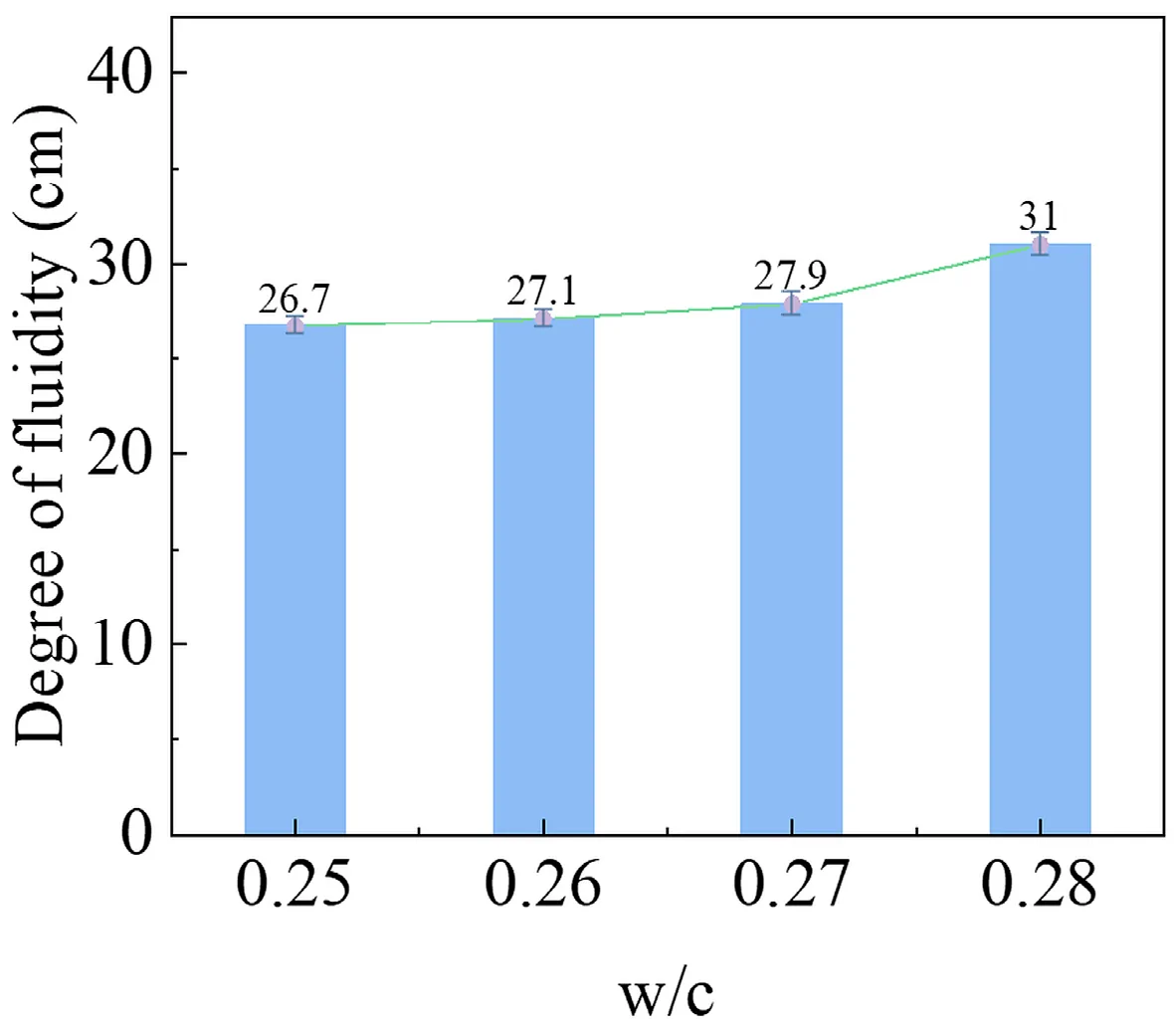
Fluidity of ASWGM under different W/C.

The test results of fluidity under different W/C are shown in Figure 13. Obviously, the fluidity of grouting materials increased by 46.5% from 0.24 to 0.28 with the increase in the W/C. The fluidity of the slurry rose significantly as the W/C increased. The slurry with a higher W/C had a greater free water content, thereby increasing the free water content in the pores. The frictional force of different aggregations in the slurry decreased, thus significantly increasing the initial fluidity.

### 3.4. Impact of Hydration Time on the Rheological Properties of ASWGM

**Figure 14:**
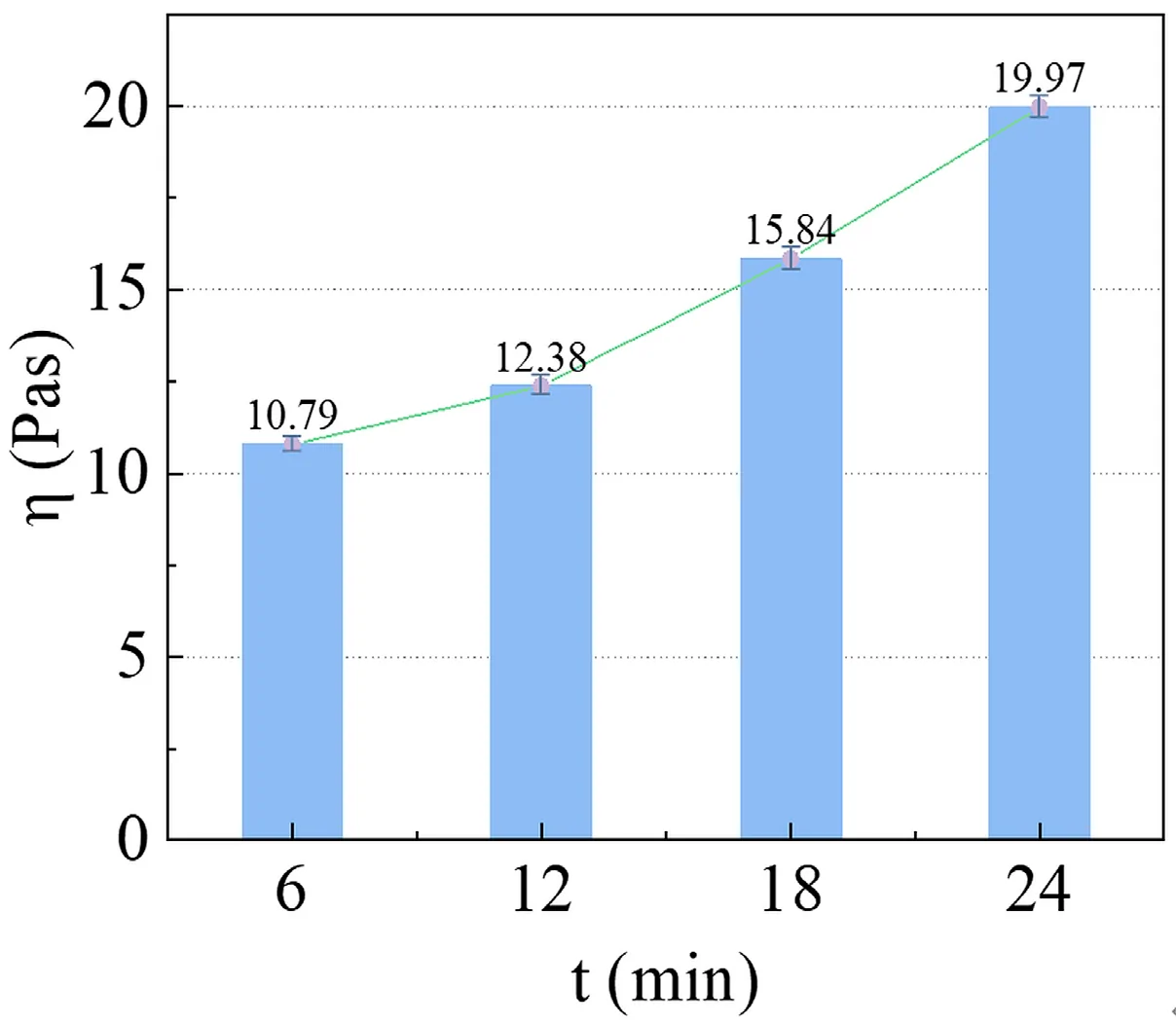
Apparent viscosity of ASWGM under different hydration times.

The fluidity of grouting materials gradually declined as hydration time increased. The impact of hydration time on fluidity was further investigated and determined. The viscosity variation pattern of the grouting materials under different hydration times and the same shearing rate is shown in Figure 14. Clearly, the viscosity slightly increased within 15 min before the hydration, and the apparent viscosity at 24 min of hydration increased by 80.76% compared to that at 6 min of hydration. The increasing trends of viscosity after 18 min of hydration are obvious. This is because the slurry becomes increasingly compact as the hydration continues to increase the number of flocculent structures in the slurry. In addition, some free water can be changed into chemically bound water, thus increasing the frictional force among the particles in the slurry and among the flocculent structures, leading to enhanced apparent viscosity.

**Figure 15:**
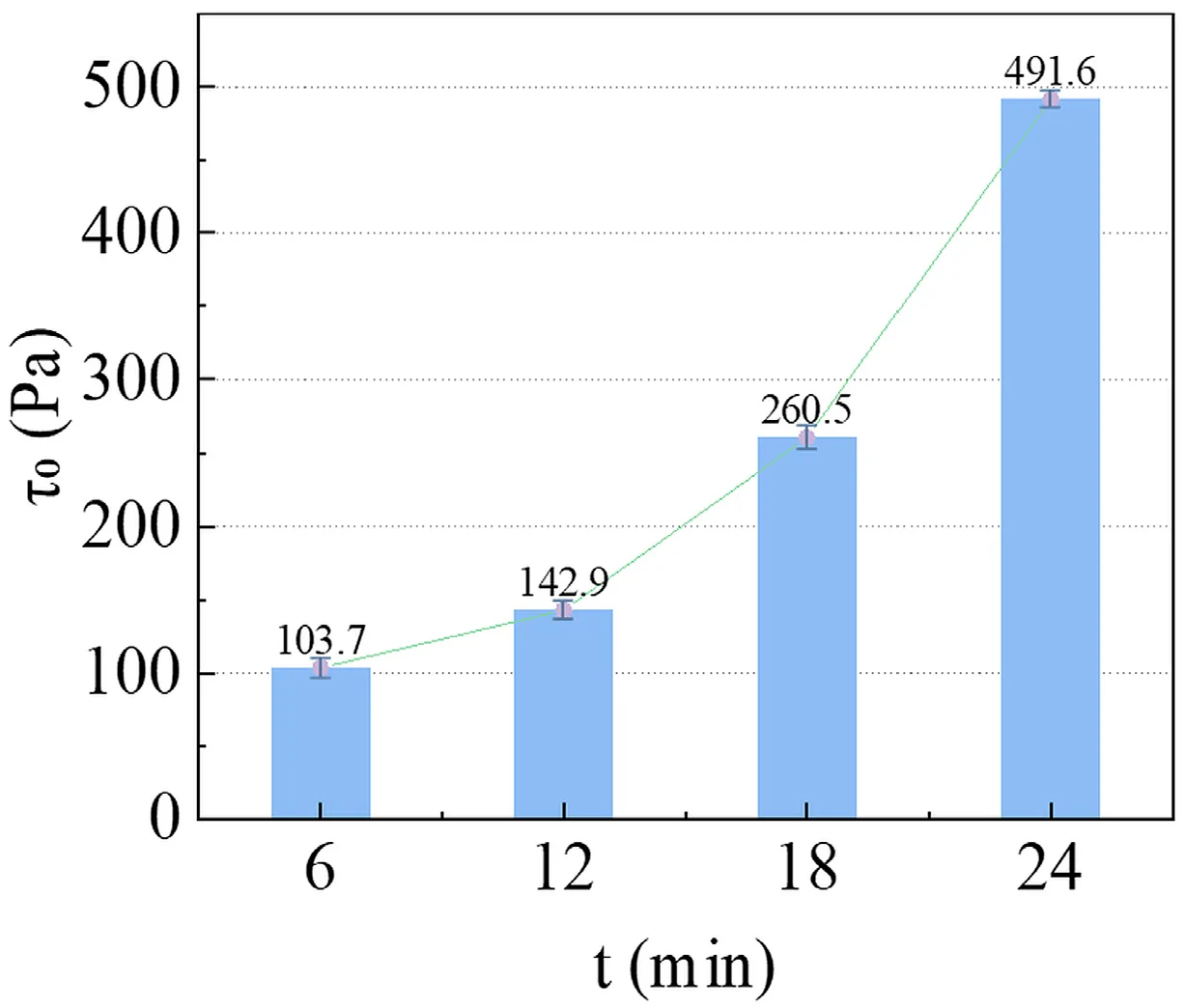
Yield stress of ASWGM under different hydration times.

The yield stress fitted from the H–B model under different hydration times is shown in Figure 15. Over time, the yield stress of the slurry exhibited a corresponding rise. The particles in the fresh slurry repel one another due to the effects from various ions in the additives, thus resulting in the low yield stress during the early stage. The stress greatly increased after 18 min. This is because the substrate of cementing materials involves early strength substances that can react with water to generate abundant hydration products. The produced hydration products are continuously gathered on the flocculent structure, forming clusters and increasing the retardation of the rotor. Hence, 18 min was determined as the test period of grouting materials.

Mechanistically, the viscosity and yield stress changes observed in Section 3.1, Section 3.2, Section 3.3 and Section 3.4 are governed by four synergistic effects induced by silica fume: (1) particle-packing effect, where ultra-fine silica fume particles (<1 μm) fill the interstitial voids between larger solid waste particles, releasing bound capillary water; (2) interparticle forces, where the high specific surface area of silica fume enhances colloidal attractive forces and interparticle friction, increasing early yield stress and shear-thinning response; (3) nucleation effect, where distributed silica fume nanoparticles act as nucleation sites that accelerate early hydration precipitation; and (4) pozzolanic reaction, where silica fume rapidly consumes free Ca(OH)_2_ to form C-S-H gel networks, promoting early structural build-up [42,43].

### 3.5. Compressive Strength Analysis of ASWGM

**Figure 16:**
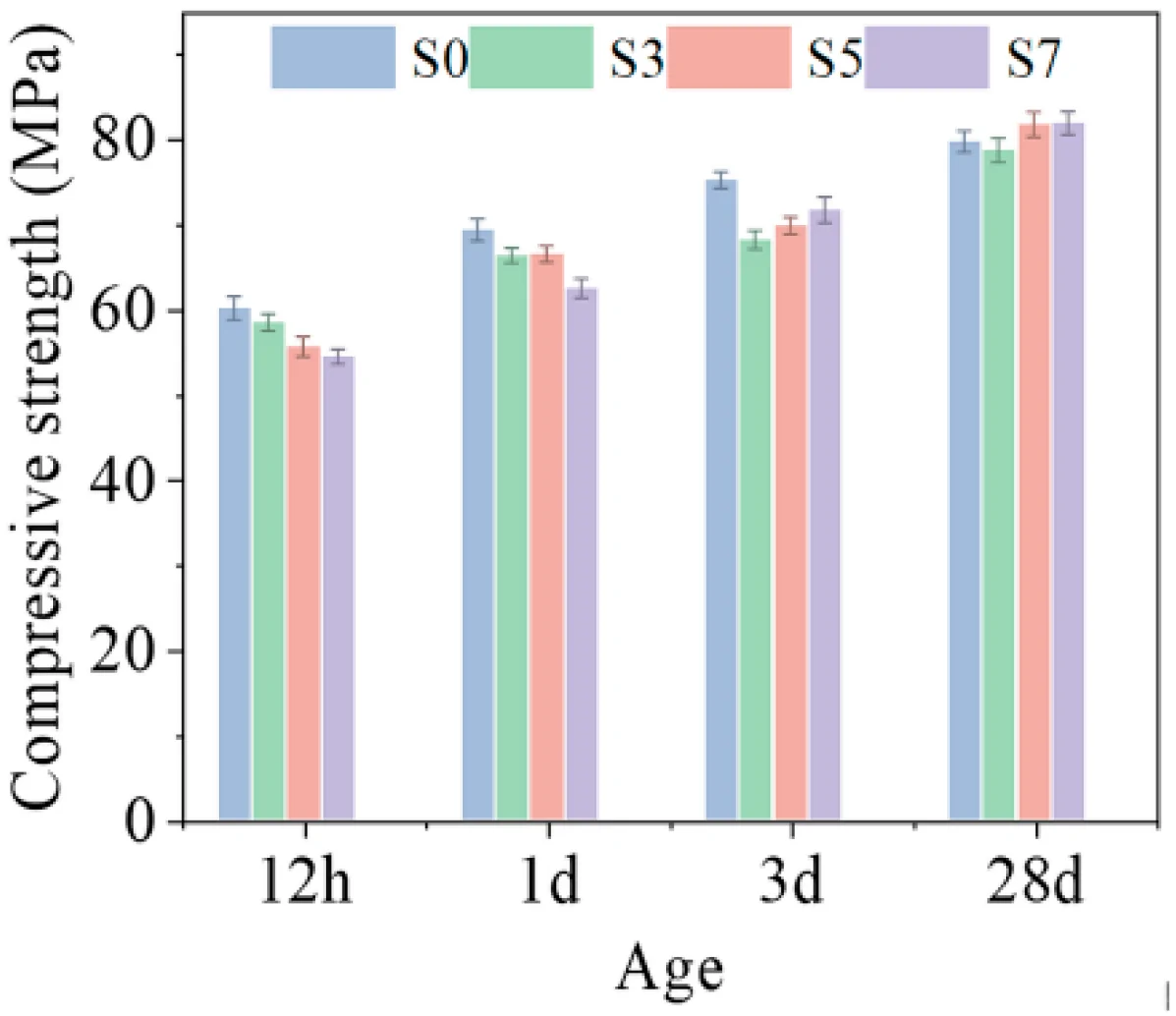
Compressive strength of ASWGM under different silica fume contents.

Grouting materials exhibiting both good fluidity and compressive strength are considered optimal. The compressive strength of grouting materials containing varying silica fume contents was analyzed, with the results presented in Figure 16. Obviously, the early strength (12 h) gradually declined with the increase in the silica fume content. The compressive strength of S0, S3, S5, and S7 was 60.2 MPa, 58.4 MPa, 55.6 MPa, and 54.5 MPa, respectively. The late strength (28d) was positively related to the silica fume content. The compressive strength of S0, S3, S5, and S7 was 79.8 MPa, 78.8 MPa, 81.7 MPa, and 82.0 MPa, respectively. In summary, the grouting materials develop notable early strength, with the average compressive strength at 12 h reaching approximately 70% of that at 28d. This is applicable to some fast construction scenes. The strength of S5 with good fluidity uniformly increased and its late strength was close to that of S7. Hence, it is reasonable to study the fluidity of S5.

**Figure 17:**
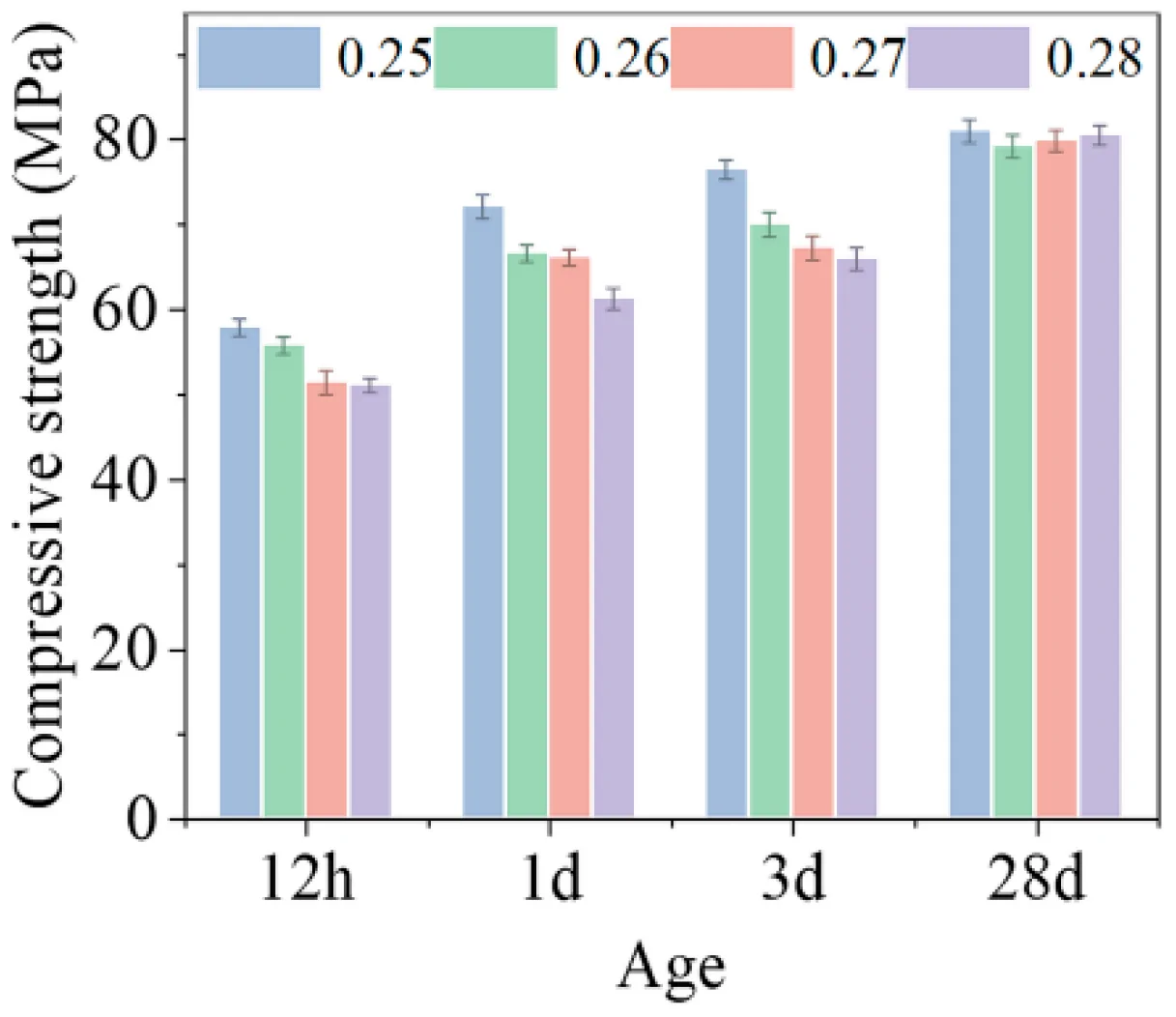
Compressive strength of ASWGM under different W/C.

The variation laws of the compressive strength of grouting materials under different W/C are shown in Figure 17. Clearly, the strength at 0.25 was better than that at 0.26 or higher, especially within 3d. As can be seen, a low W/C can greatly increase the early and overall compressive strength of the grouting materials. The early strength was slightly low when the W/C was higher, but the later strength is almost the same as that of the low W/C. Hence, different W/C can be chosen according to the needs of fluidity. The compressive strength of grouting materials exceeded 50 MPa at 12 h. The optimal fluidity was chosen as the W/C is 0.28 and the strength was also good.

### 3.6. Chemical Substance Analysis of ASWGM

**Figure 18:**
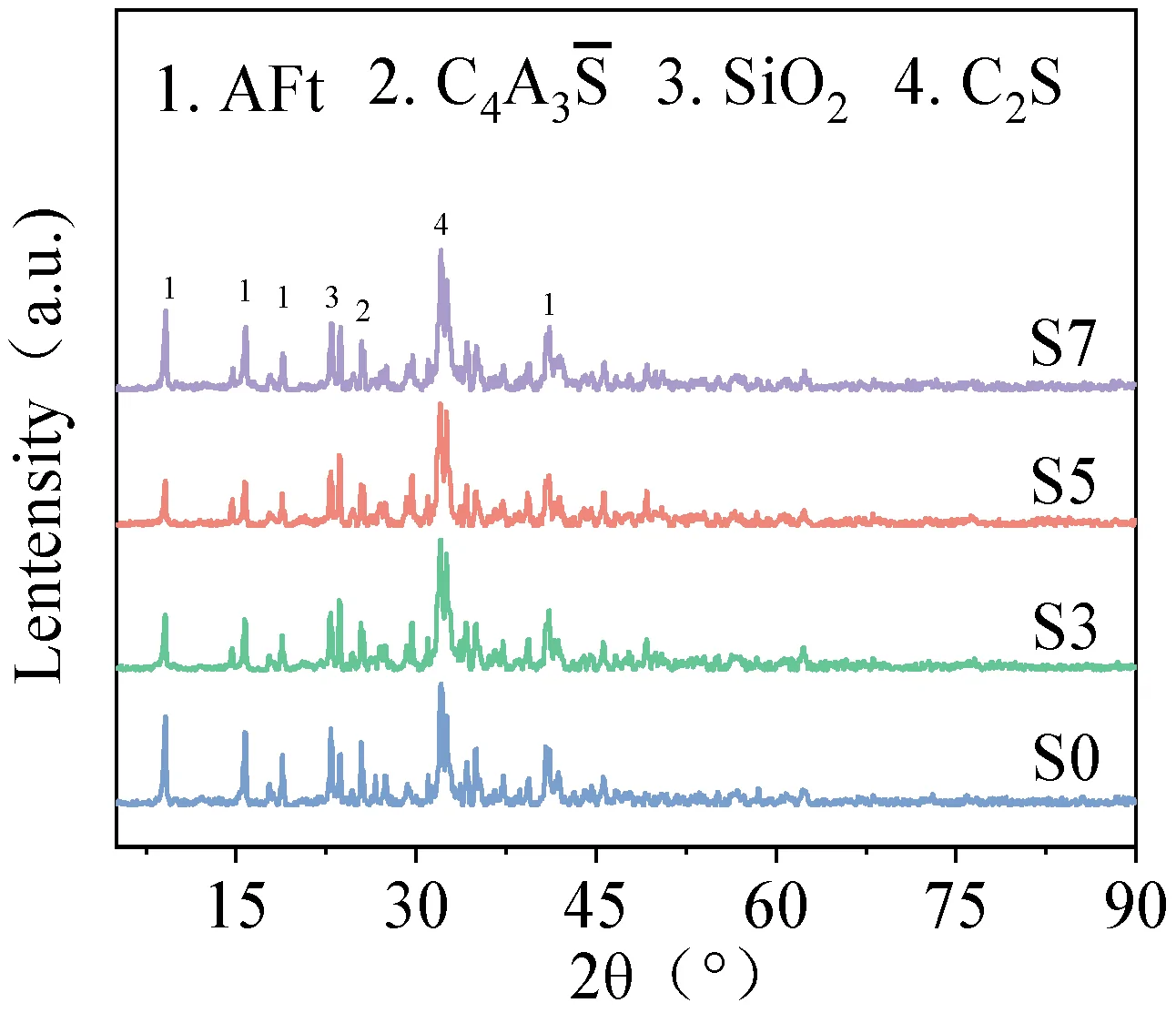
XRD spectra of ASWGM under different silica fume contents.

**Figure 19:**
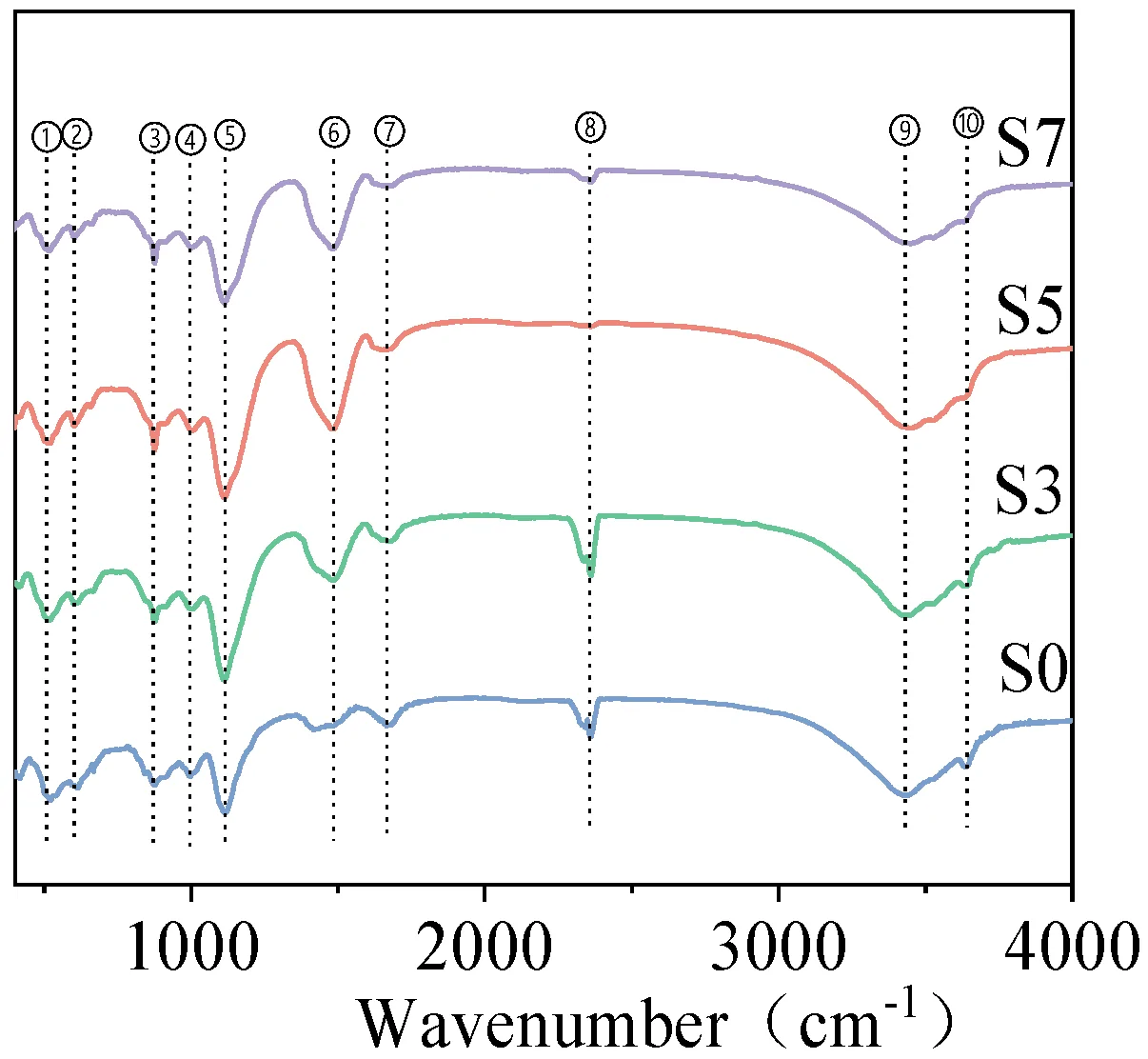
Infrared spectroscopy of ASWGM under different silica fume contents.

The XRD phase analysis diagram under different silica fume contents is shown in Figure 18. Clearly, adding silica fume may not change the types of hydration products, which are AFt, C_4_A_3_$\overset{-}{S}$, SiO_2_ and C_2_S. As the silica fume content increases, the diffraction peak intensity of ettringite reaches its maximum at S0. Combining with the compressive strength, the ettringite content determines the strength of the grouting materials. The infrared spectrogram at the same time is shown in Figure 19. A stretching vibration peak of Si-O and a stretching vibration peak of O-H at ③, ⑥ and ⑨ were detected, respectively, indicating the existence of AFt. The stretching vibration of O-H proves the existence of Ca (OH)_2_ in the slurry. According to the XRD analysis results, the Ca(OH)_2_ content significantly decreased with the increase in the silica fume content. Specifically, the diffraction peak intensity of Ca(OH)_2_ when the silica fume content was 3% and 5% decreased by 91.92% and 80.67% compared to that when silica fume was not added. This result reflects that there is a chemical reaction between the silica fume and Ca(OH)_2_, thus consuming Ca(OH)_2_. This result demonstrates that fine particles of silica fume serve as a crystal nucleus core during cement hydration and facilitate cement hydration. The dual influence of silica fume on early rheology and later strength stems from its physical–chemical role during hydration. Physically, ultra-fine silica fume fills interparticle voids and releases trapped water to improve early fluidity. Chemically, it provides heterogeneous nucleation sites to accelerate early hydrate precipitation, while its active SiO_2_ rapidly consumes Ca(OH)_2_ through secondary pozzolanic reactions. This transforms loose CH into dense C-S-H gel that interlocks with early ettringite (AFt) needles, achieving rapid early strength (>50 MPa at 12 h) and significant late-stage densification (>80 MPa at 28d).

**Figure 20:**
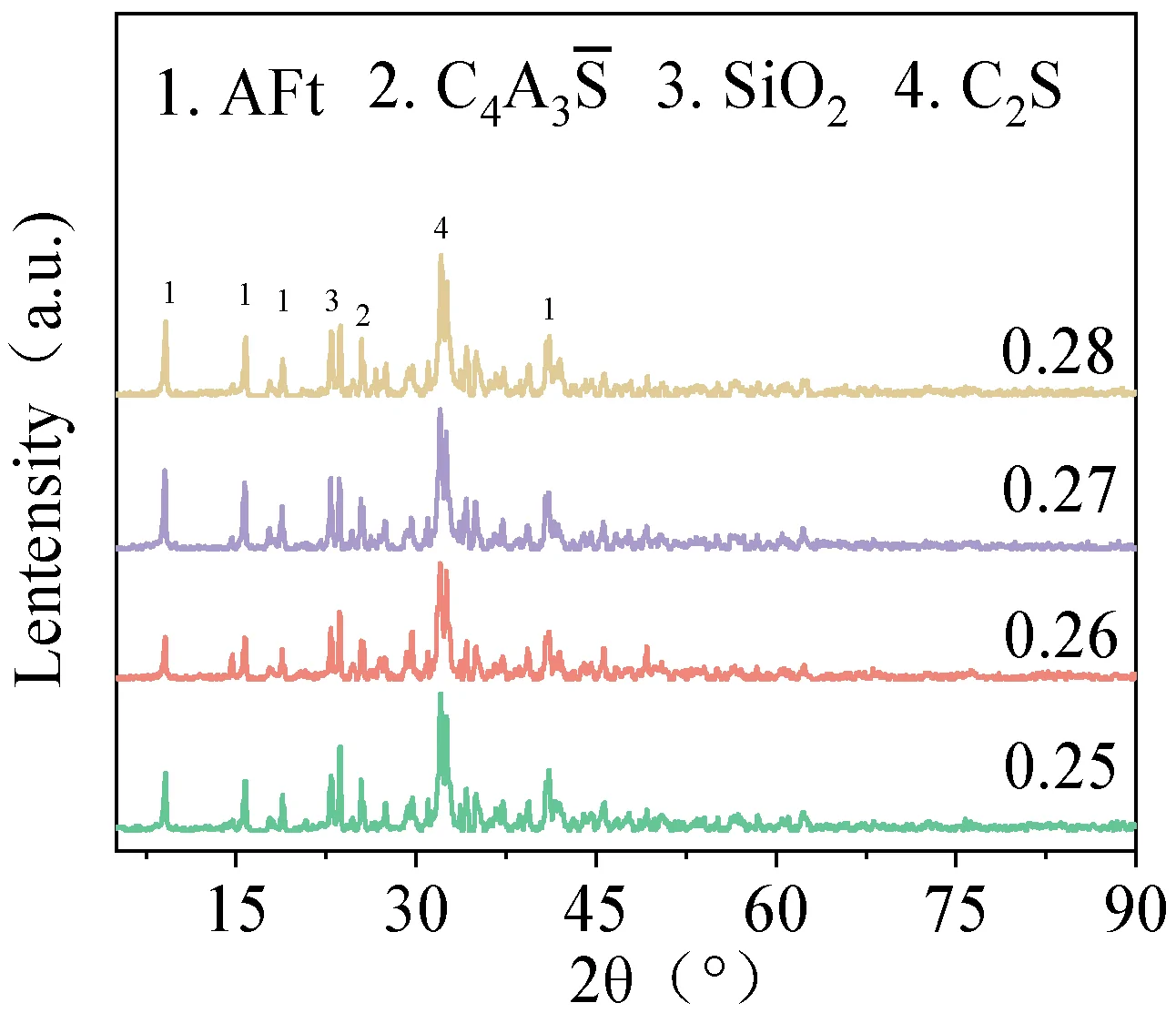
XRD of ASWGM under different W/C.

**Figure 21:**
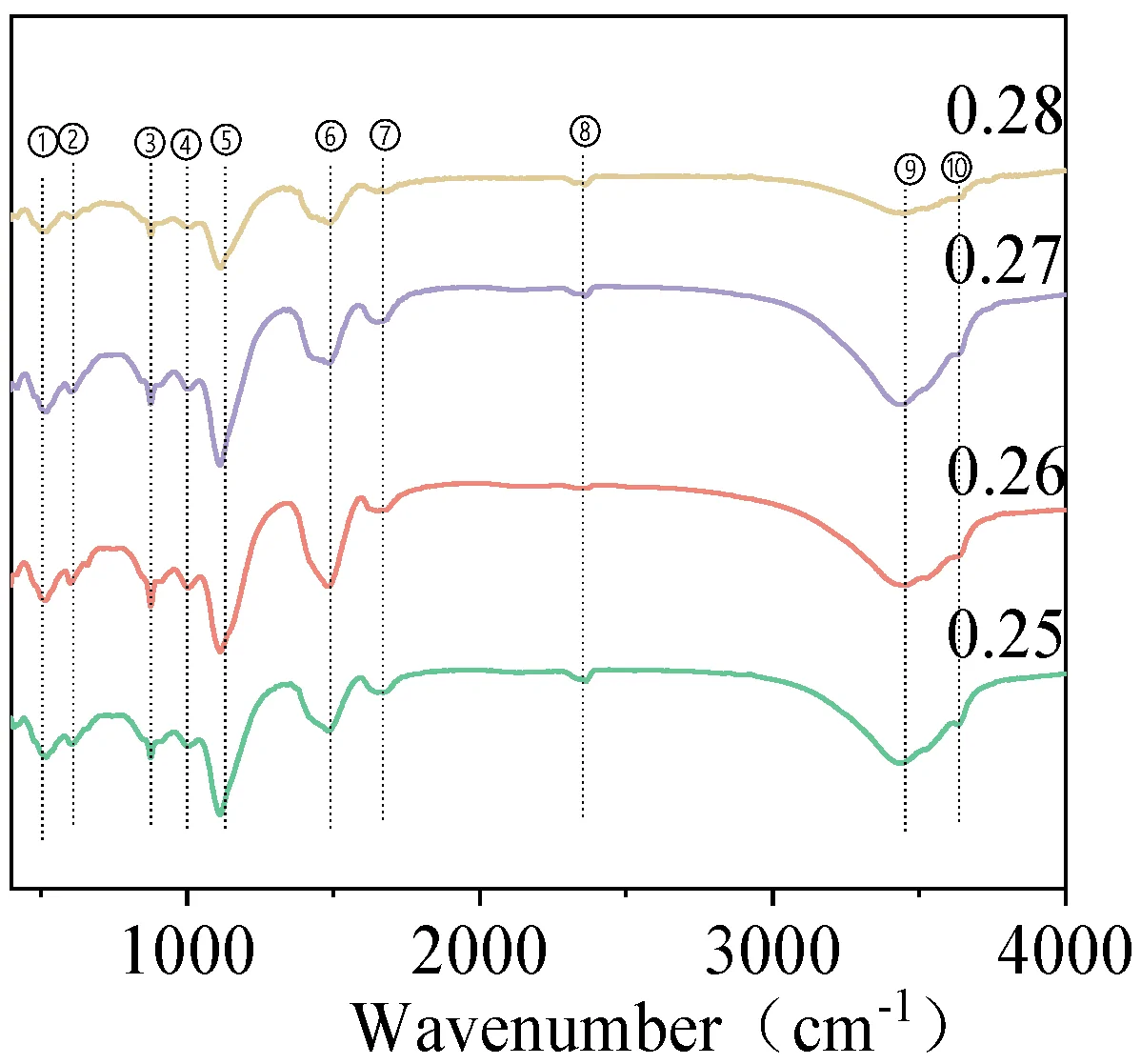
Infrared spectroscopy of ASWGM under different W/C.

As shown in Figure 20 and Figure 21, at 3d of hydration, there are tremendous residues of solid waste phases (C_2_S and C_4_A_3_S) in the grouting materials with a low W/C (0.24–0.26), accompanied by a gradual growth in the production of AFt ettringite and C-S-H gels. For grouting materials with a W/C of 0.26, the AFt peak intensity reached its maximum at 3 days, and the resulting dense structure contributed to a high compressive strength of 76.37 MPa. Meanwhile, grouting materials with a W/C of 0.27 produced the highest amounts of AFt and C-S-H. In their XRD patterns, the peaks of unreacted C_2_S and C_4_A_3_S phases nearly vanished, accompanied by a distinct elevation in the baseline of amorphous C-S-H, indicating optimal microstructural compactness. The corresponding compressive strength was 67.2 MPa. Due to the free water dilution effect of the grouting materials with a high W/C, the AFt production was inhibited and there were loose C-S-H structures. Furthermore, C-H (Ca(OH)_2_) enriched and the compressive strength greatly declined to 65.8 MPa. When the W/C was 0.27, the vibration peak of the Si-O bond was the lowest, indicating the highest polymerization degree of the C-S-H gel and compact silica network. When the W/C was 0.28, the lower peak width increased, reflecting the loosening structures of the C-S-H gels. This is consistent with the reduction in the mechanical strength. In the grouting material with a W/C of 0.28, the C-H peak and CO_3_^2−^ peak were strengthened, indicating the decreased alkalinity of the pore solution and carbonization tendency. This leads to a further weakening of the interface bonding force. When the W/C was 0.25–0.26, the compressive strength reached the peak as a response to the collaborative effect of AFt-dominated early strengthening effect. When the W/C was 0.27–0.28, the crystallinity of the hydration products decreased due to the free water dilution. The enrichment of C-H and the increase in porosity affected the material’s strength, resulting in a gradual decrease in the compressive strength.

The performance of ASWGM is governed by an integrated physical–chemical mechanism bridging particle-packing, early rheology, hydration kinetics, and mechanical strength. Physically, ultra-fine silica fume optimizes the grain size distribution toward an ideal packing density, releasing trapped pore water and producing a ‘ball-bearing’ lubrication effect that drastically lowers initial yield stress and enhances fluidity. Chemically, silica fume acts as crystal nuclei during early hydration, where rapid pozzolanic reaction with Ca(OH)_2_ released from carbide slag and coal gangue forms primary C-S-H gel and ettringite (AFt). This hydrate formation converts free water into bound water, inducing time-dependent viscosity growth and thixotropic structural recovery. Concurrently, the early AFt network provides rapid early strength (>50 MPa at 12 h), while the continuous precipitation of highly polymerized C-S-H densifies the micro-pore structure, ultimately achieving superior long-term mechanical performance (>80 MPa at 28d).

Although microstructural quantification tools such as TG-DTG and MIP were not applied in this initial screening study, earlier findings on similar solid-waste systems [44,45] confirm that active silica fume accelerates Ca(OH)_2_ consumption to form gel phases and fills capillary voids, thereby converting macropores into gel pores and promoting densification.

### 3.7. Practical Application Scenarios and Performance Comparison

Featuring low initial yield stress (126.4 Pa), rapid dynamic fluidity (31 cm), exceptional early strength (>50 MPa at 12 h), and high late strength (>80 MPa at 28 d), the optimized ASWGM is particularly suited for fast-turnaround engineering applications, such as desert photovoltaic foundation anchor grouting, long-distance mine backfilling, and rapid underground structural repairs [1]. Compared with conventional commercial cement-based non-shrink grouts (which typically rely on energy-intensive cement clinker and exhibit 12 h strengths of only 20–30 MPa), ASWGM utilizes 100% industrial solid wastes (coal gangue, carbide slag, and steel slag sand). This formulation cuts embodied carbon emissions by over 70% and material costs by 30–40% while exceeding commercial products in both early load-bearing capacity and long-term mechanical durability (>80 MPa at 28d) [11,29], demonstrating superior economic and environmental viability for sustainable engineering practice.

It should be noted that a single-factor sequential design was adopted here to isolate the fundamental micro-mechanisms of individual variables on the cement-free matrix. Consequently, the parameters (5% silica fume, 20 °C, and W/B of 0.28) reflect the best-performing engineering combination within the tested ranges rather than a mathematically strict global optimum. Future work using multi-variable statistical optimization (e.g., response surface methodology) is recommended to explore coupled interaction effects.

## 4. Conclusions

In this study, the performance of an all-solid-waste grouting material (ASWGM) was systematically evaluated under specified laboratory conditions. Based strictly on the empirical findings and physical–chemical characterizations, the main conclusions are drawn as follows:

1. Rheological optimization within tested limits: Within the experimental ranges investigated, incorporating 5 wt% silica fume (S5) at a W/C ratio of 0.28 and an ambient temperature of 20 °C provided the most favorable balance of workability for the tested mixtures, yielding an initial fluidity of 31 cm and a yield stress of 126.4 Pa within an 18 min elapsed operational window.
2. Structure–property correlations: A strong interrelationship was identified between initial particle dynamics, early hydration, rheology, and mechanical performance. Ultra-fine silica fume particles (<1 μm) refined the overall particle size distribution and acted as nucleation sites, which increased initial yield stress and shear-thinning response. Concurrently, the accelerated formation of early C-S-H gel networks directly underpinned the high early compressive strength (>50 MPa at 12 h and >80 MPa at 28 d).
3. Empirical mechanistic link: Spectroscopic (FTIR) and diffraction (XRD) analyses confirm that the pozzolanic consumption of free Ca(OH)_2_ by active silica fume accelerates gel precipitation. This microstructural densification directly explains the observed transition from initial fluid-like slurry behavior to rapid matrix strength gain, without extrapolating beyond the scope of the present dataset.

## Figures and Tables

**Table 1:** Main chemical components of raw materials (%).

	CaO	SiO_2_	Al_2_O_3_	Fe_2_O_3_	SO_3_	MgO	TiO_2_
Calcium carbide slag	78.66	11.5	3.647	0.124	1.832	1.623	0.134
Coal gangue	6.143	41.843	45.604	0.735	1.343	1.216	1.152
Desulfurization gypsum	28.438	9.107	0.665	/	51.257	4.217	/
Fly ash	16.243	46.869	21.406	6.199	1.346	2.308	0.672
Silica fume	0.873	83.139	4.395	2.266	0.533	1.621	0.101

**Table 2:** Performance index of the steel slag sand.

Aggregate Type	Bulk Density kg/m^3^	Specific Surface Area m^2^/g	Apparent Density kg/m^3^	Water Absorption Rate %
Steel slag sand	2020	0.0065	3750	0.5

**Table 3:** Test mixing ratio of grouting material with different silica fume contents.

No.	Silica Fume/g	Solid-Waste Binder/g	Fly Ash/g	Steel Slag/g	Water/g	Retarder/g	Water Reducer/g
S0	0	500	50	550	143.0	2.75	0.825
S3	15	500	50	565	146.9	2.825	0.8475
S5	25	500	50	575	149.5	2.875	0.8625
S7	35	500	50	585	152.1	2.925	0.8775
0.25	25	500	50	575	143.7	2.875	0.8625
0.26	25	500	50	575	149.5	2.875	0.8625
0.27	25	500	50	575	155.2	2.875	0.8625
0.28	25	500	50	575	161.0	2.875	0.8625

Note: S0, S3, S5, and S7 designate formulations with silica fume added at 0 wt%, 3 wt%, 5 wt%, and 7 wt% by mass of the total solid binder, respectively (e.g., S0 denotes the baseline control mix with 0 wt% silica fume).

**Table 4:** Physical properties of ASWGM.

Type	Expansion Rate	Water Requirement of Normal Consistency	Initial Setting Time	Final Setting Time	Stability	Compressive Strength
Type III	0.18	0.26	37 min	1 h 6 min	Qualified	75.3

## Data Availability

The original contributions presented in this study are included in the article. Further inquiries can be directed to the corresponding author.
